# Coupling inertial, viscoelastic, and enhanced secondary flow in a composite microchannel: achieving high-precision multi-sized particle 3D central co-focusing

**DOI:** 10.1038/s41378-026-01254-9

**Published:** 2026-04-15

**Authors:** Tianwei Zhao, Peng Zeng, Chenchen Ji, Xu Yin, Jinxia Li, Xing Chen, Yuanming Ma, Gaobin Xu, Xichen Yuan, Jianguo Feng

**Affiliations:** 1 School of Microelectronics, Hefei University of Technology, Hefei, Anhui 230601 China; 2 School of Mechanical Engineering, Northwestern Polytechnical University, Xi’an, Shaanxi 710072 China; 3 Department of Medical Laboratory, Xi’an International Medical Center Hospital, Xi’an, Shaanxi 710100 China; 4 Department of Microsystems Engineering, University of Freiburg, Freiburg, 79110 Germany

**Keywords:** Engineering, Chemistry, Physics

## Abstract

Microfluidic particle focusing is essential for diverse biomedical applications. However, conventional inertial focusing techniques are limited by particle size dependency, hindering effective 3D central co-focusing of particles with varying sizes. In this study, we introduced a novel microfluidic method based on an inertial-viscoelastic-secondary flow synergistic effect (INVEST) within a composite microchannel (CMC), enabling high-efficiency 3D co-focusing of multi-sized particles. The CMCs incorporated height-varying horizontal and vertical semicircular obstacles to modulate inertial and secondary flows, while hyaluronic acid (HA) was introduced to enhance the viscoelastic effect and balance the force disparities among particles. Comprehensive numerical simulations were conducted to analyze the main flow field, secondary flow vectors, and shear-rate distributions. A novel metric, equilibrium zone width (EZW), was first proposed to theoretically assess the focusing performance. The simulation results indicated a minimal EZW of 15.58 μm. Moreover, experimental validations across various HA concentrations, obstacle configurations, and particle sizes demonstrated focusing widths below 20.5 μm and efficiencies exceeding 95% for particle mixtures with diameters from 10 to 20 μm. Further testing using white blood cells confirmed a focusing efficiency of 96.14%. These findings verified that the CMCs successfully integrated inertial migration, viscoelastic effects, and enhanced secondary flows to realize the INVEST mechanism within a single microchannel, effectively addressing the issues of size dependency of traditional inertial focusing and the corner attraction effect of viscoelastic focusing. The developed microfluidic platform enables robust 3D central co-focusing of multi-sized particles and heterogeneous cells, providing a promising solution for high-throughput microflow cytometry and single-cell analysis.

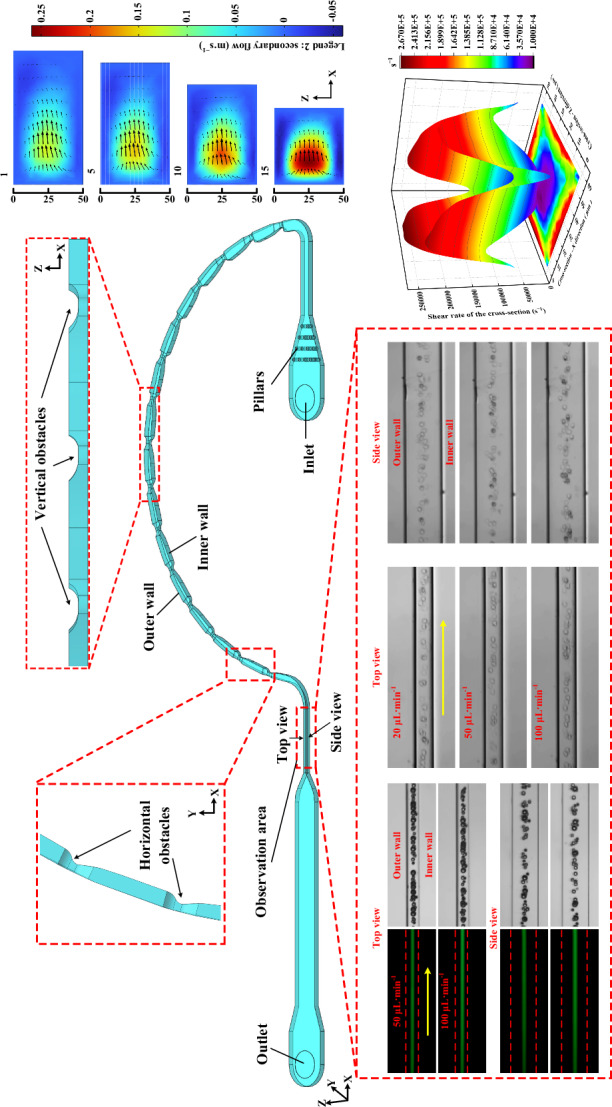

## Introduction

Microfluidic particle manipulation techniques, including focusing, separation, enrichment, filtration, extraction, and sorting, serve as fundamental tools in extensive applications, such as biochemical analysis, food and environmental testing, sample pretreatment, and medical diagnostics^1–5^. Among them, the particle focusing technique is a critical prerequisite for achieving high-precision, high-efficiency, and high-throughput particle manipulation. Inertial focusing has emerged as a powerful method because of its simple device fabrication, ease of integration, minimal particle damage, high throughput, and low cost. It has been widely used in single-cell manipulation and analysis. High-precision 3D co-focusing of multi-sized particle mixture and cells plays a critical role in a broad range of downstream cell analysis applications, including microflow cytometry, electrical impedance sensing, and optical detection. By guiding particles and cells of different sizes to a single, well-defined 3D streamline, all analytes experience identical optical illumination, electric field distribution, and hydrodynamic shear conditions during interrogation. This unified flow environment significantly enhances signal uniformity, reduces measurement variance, and improves quantitative accuracy, thereby ensuring that observed signal differences originate from intrinsic cellular properties rather than positional or trajectory-dependent artifacts.

Traditional inertial focusing relies on fluid inertial effect and secondary flow, achieving particle focusing through the dynamic equilibrium of the inertial lift force and Dean drag force^6–9^. For instance, Xiang et al. developed a five-loop spiral device that enabled tight focusing of 4.8 μm particles near the inner wall^10^. Zhang et al. reported a square-wave serpentine channel design that achieved single-stream focusing in symmetric serpentine channels^11^. Kuntaegowdanahalli et al. demonstrated that larger particles were focused to the equilibrium position near the inner wall, whereas smaller particles were closer to the channel center^12^. The strong size dependence of this technique makes it challenging to achieve 3D co-focusing for multi-sized particles.

Recent advances in viscoelastic microfluidic technology have opened new avenues for particle manipulation^13^. By introducing polymer additives into fluids, the viscoelastic properties of the fluid induce additional elastic forces orthogonal to the inertial lift forces, enabling 3D particle focusing. Under conditions of negligible inertia, Leshansky et al. observed that particles migrated toward the centerline and achieved focusing due to the first normal stress difference gradient^14^. However, Yang et al. demonstrated that in rectangular channels, elastic lift forces drive particles toward both the centerline and corners, achieving two-dimensional focusing, whereas effective 3D focusing fails to be established at the channel center^15^. Additionally, viscoelastic focusing techniques also suffer from sensitivity to polymer concentration and low throughput^16–18^.

Based on our previous research on hybrid microchannels with variable cross-sections^19^, this study proposed a microfluidic particle focusing method using an inertial-viscoelastic-secondary flow synergistic effect (INVEST) generated in a composite microchannel (CMC) for high-efficiency 3D co-focusing of multi-sized particles. We presented multidimensional composite microchannels by introducing a series of semicircular obstacles in both horizontal and vertical directions to synergistically regulate the intensity and distribution of secondary flows. Horizontal obstacles modulate the lateral velocity gradients and suppress size-dependent migration by redistributing elastic stresses across the channel width, while vertical obstacles generate controlled vertical secondary flows and suppress corner attraction, enabling particles that would otherwise migrate toward the top or bottom walls to be driven back toward the central plane. Concurrently, hyaluronic acid (HA) was used in samples to induce elastic forces. Combined with the inertial lift force and secondary flow drag forces, different size particles were equilibrated at the same position, thereby facilitating the 3D co-focusing of multi-sized particles.

In order to verify our INVEST-based particle focusing method, theoretical simulations were first performed to quantify the main flow fields, secondary flow vectors, and shear-rate distributions inside CMCs. A novel metric, equilibrium zone width (EZW), was proposed to theoretically assess the focusing performance of the microfluidic devices. Then, we utilized 15 μm particles to characterize the concentration of HA solutions and two types of CMC configurations (in-phase and out-of-phase vertical/horizontal obstacle configurations). Focusing experiments with different types of particles and particle mixtures were also conducted to test the focusing performance of our devices. Finally, we carried out the white blood cells (WBCs) focusing experiments, demonstrating that the proposed INVEST-based CMCs enabled single-sized particle focusing, multi-sized particle co-focusing, and biological cell focusing, offering a significant role in microflow cytometry and single-cell analysis.

## Theory

### Inertial particle focusing

The inertial magnitude of a fluid is commonly described by the dimensionless Reynolds number (Re), defined as the ratio of the inertial lift force to the viscous force^20^:

1 $${\mathrm{Re}}=\frac{{\rho {UD}}_{{\rm{h}}}}{\mu }$$

where *ρ* is the fluid density, ${U}$ is the fluid velocity, μ is the dynamic viscosity, and *D*_h_ is the hydraulic diameter. For rectangular channels, *D*_h_ is calculated as 2*wh*/(*w* + *h*)^21,22^, where *w* and *h* represent the width and height of the channel cross-section, respectively.

Traditional microfluidics assumes Stokes flow, where particles follow the main flow direction without lateral migration. The discovery of inertial migration by Segre and Silberberg revealed that particles in circular channels migrate to an annular equilibrium position at 0.6 times the channel radius^23^. Subsequent studies have established that particles in microchannels experience transverse forces dominated by two lift forces: the shear-gradient lift force (*F*_S_), directed toward the channel wall due to parabolic velocity gradients in Poiseuille flow, and the wall-induced lift force (*F*_W_), directed toward the channel centerline due to particle-wall interactions. The resultant force is termed the net inertial lift force (*F*_L_)^20,24–26^:

2 $${\rm{F}}_{\rm{L}}=\frac{{f}_{L}{\rho {U}^{2}}{a}^{4}}{D\frac{2}{{\rm{h}}}}$$

where *a* is the particle diameter, and *f*_L_ is a dimensionless lift coefficient.

Secondary flows play a critical role in reducing the equilibrium positions and adjusting the particle focusing locations. In curved channels, a pressure gradient from the inner to the outer region generates two counter-rotating vortices perpendicular to the main flow. The Dean number (De) characterizes the vortex intensity^27^:

3 $${\rm{De}}={\rm{Re}}\sqrt{\frac{{D}_{\rm{h}}}{{2}{R}}}$$

where *R* is the radius of curvature. Dean vortices exert Dean drag forces (*F*_D_) on the particles^27–29^:

4 $${F}_{{\rm{D}}} \sim \frac{{{\mu }^{2}a\mathrm{De}}^{2}}{{\rho D}_{{\rm{h}}}} \sim \frac{{{\mu }^{2}a\mathrm{Re}}^{2}}{2\rho R}$$

Additionally, the introduction of obstacles on the walls of microchannels can cause variations in the channel cross-section, which generates secondary flows, referred to as geometry-induced secondary flows or Dean-like flows.

### Viscoelastic particle focusing

The viscoelasticity of a fluid is quantified by the Weissenberg number Wi^30^:

5 $${\rm{Wi}}=\lambda \dot{\gamma}=\lambda \frac{2U}{{D}_{{\rm{h}}}}$$

where *λ* is the relaxation time of the polymer solution and $\dot{\gamma}={2U/D}_{{\rm{h}}}$ is the characteristic shear rate. When Wi ≫ 1, the fluid primarily exhibits elastic behavior, resembling solid properties, whereas when Wi ≈ 0, the fluid primarily exhibits viscous behavior, resembling liquid properties^31,32^.

Particle migration in viscoelastic fluids is governed by the elastic forces directed toward low-shear-rate regions. Microscopically, particles experience normal stress differences: the first normal stress difference (*N*_1_=*σ*_*xx*_*-σ*_*yy*_) and the second normal stress difference (*N*_2_=*σ*_*yy*_*-σ*_*zz*_). For most viscoelastic fluids, *N*_2_ ≈ 0.1*N*_1_, and is often neglected. The elastic force (*F*_E_) is approximated as^13^

6 $${F}_{{\rm{E}}}={{C}_{{\rm{E}}}a}^{3}{\nabla N}_{1}={{C}_{{\rm{E}}}a}^{3}\left({\nabla {\rm{\sigma }}}_{{xx}}-{\nabla {\rm{\sigma }}}_{{yy}}\right)={{{-2C}_{{\rm{E}}}a}^{3}{\eta }_{{\rm{p}}}{\rm{\lambda }}\nabla \dot{\gamma }}^{2}$$

where *C*_E_ is the non-dimensional elastic lift coefficient and *η*_p_ is the polymeric contribution to the solution viscosity.

### Inertial-elasto-secondary flow particle focusing

**Fig. 1:**
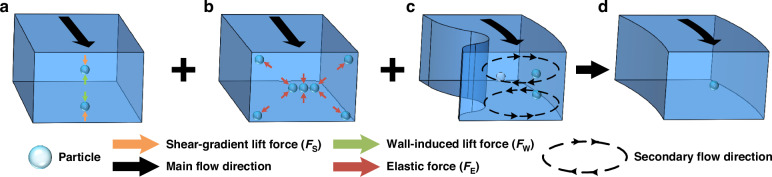
Schematic illustration of the synergistic effect (INVEST) of fluid inertia, viscoelasticity, and secondary flow From left to right: particle distributions and force profiles under **a** inertial effect, **b** viscoelastic effect, **c** secondary flow effect, and **d** INVEST effect. The blue spheres represent the particle equilibrium positions. The black solid arrows indicate the main flow direction. Three colored solid arrows denote the shear-gradient lift force (orange), wall-induced lift force (green), and elastic force (red), with the arrow orientations indicating the force directions. Dashed arrows indicate the direction of the secondary flow

In this work, we demonstrated that 3D particle focusing can be achieved in a composite channel based on INVEST. For viscoelastic fluids, both Wi and Re increase with increasing flow velocity. Particle migration is determined by the coupling and competition of three forces: *F*_L_, *F*_E_, and *F*_D_ (Fig. 1). The elasticity number (El) is an indicator of the relative importance of elasticity and inertia, and is defined as follows^13,33–36^:

7 $${\rm{El}}=\frac{{\rm{Wi}}}{{\mathrm{Re}}}=\frac{2\lambda \mu}{\rho D_{h}^{2}}$$

When El ≫ 1, particle migration is predominantly governed by the viscoelastic effect. Particles migrate toward either the channel centerline or the corner regions. Conversely, when El ≈ 0, particle migration is dominated by the inertial effect. Particles move toward the center of each wall, ultimately establishing two or four equilibrium positions.

The presence of secondary flows can suppress viscoelastic-induced corner attraction, accelerate particle migration, and can further reduce the number of equilibrium positions. In addition, as shown in Eqs. (2), (4), and (6), the net inertial lift force is positively correlated with the fourth power of the particle diameter (*F*_L_ ~ *a*⁴), while the elastic force is proportional to the third power of the particle diameter (*F*_E_ ~ *a*³), and the Dean drag force induced by the secondary flow is linearly related to the particle diameter (*F*_D_ ~ *a*). This significant disparity makes the effect of secondary flow particularly pronounced for small particles. Therefore, we employed height-varying obstacles to generate enhanced secondary flows, counteracting the equilibrium position deviation caused by differences in particle size.

We selected seven cross-sections to numerically calculate El (Table S1). The results indicated that with the INVEST configuration, elasticity and inertia were comparable in magnitude (El = 3.09–7.92). At the entrance of the CMC, where the elastic force was relatively weak, the enhanced secondary flow transported randomly distributed particles located near the channel walls and corners toward the channel center, thereby reducing the differences in equilibrium positions among particles of varying sizes. As the channel width subsequently decreased from 200 μm to 50 μm, the corresponding increase in El from 3.09 to 7.92 led to the elastic force becoming dominant. This dominant elastic force compressed the particles at the center of the channel into a single streamline, ultimately achieving high-precision 3D central co-focusing of multi-sized particles.

## Results

### Theoretical simulation of the CMCs

**Fig. 2:**
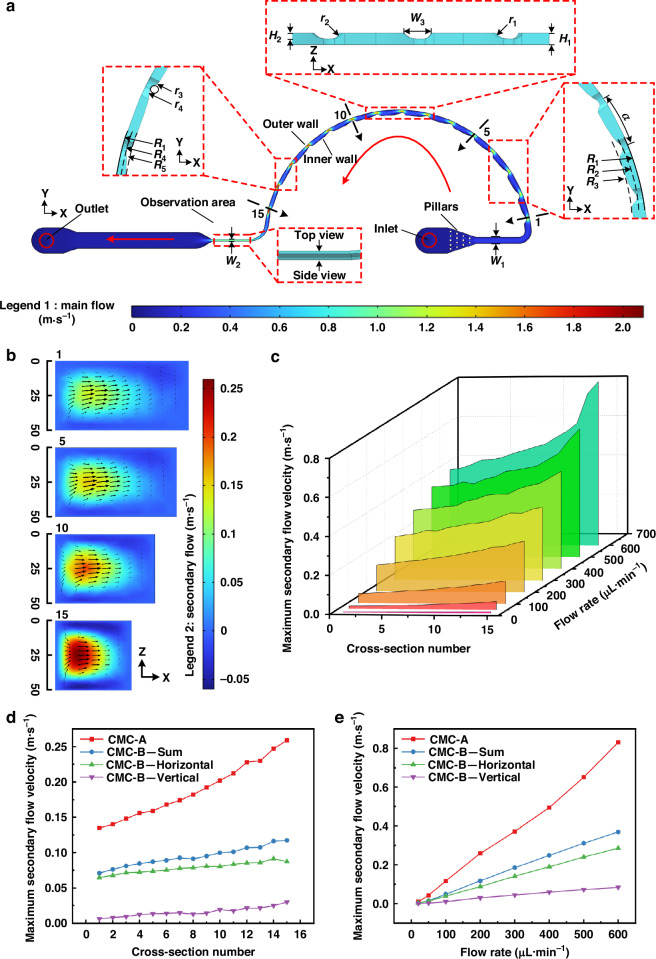
The design and simulation of CMC-A **a** Design parameters of CMC-A and color contour of the main flow velocity in CMC-A. The red arrow indicates the main flow direction, and the dashed black arrows mark the selected cross-sections. Legend 1 corresponds to the main flow velocity, ranging from 0 to 2.09 m·s^−1^. **b** Color contours of the secondary flow velocity in the four selected cross-sections of CMC-A. Black arrows indicate the secondary flow vortices. Legend 2 represents the secondary flow velocity, which ranges from −0.0541 to 0.259 ms^−1^. **c** Maximum secondary flow velocity versus cross-section number and flow rate. **d** Comparison of maximum secondary flow velocity across the cross-sections of CMC-A and CMC-B (flow rate: 200 μL·min^−1^). **e** Comparison of maximum secondary flow velocity at the 15th cross-section of CMC-A and CMC-B (flow rates: 20–600 μL·min^−1^). The simulation results for CMC-B were shown in Fig. S1. “Horizontal”, “Vertical”, and “SUM” represent the maximum secondary flow velocities near the horizontal and vertical obstacles, and the sum of the maximum secondary flow velocities near the adjacent horizontal and vertical obstacles in CMC-B, respectively

We conducted simulations of CMCs using viscoelastic solutions to evaluate the focusing performance of the two-channel structures and the effectiveness of INVEST. In the CMCs, particle dynamics were governed by the interplay of inertial lift forces, secondary flow vortices, and viscoelastic effects. The inertial lift forces and secondary flow drag forces were predominantly influenced by the main flow velocity and secondary flow intensity. To systematically investigate these effects, we simulated the flow field characteristics of two CMC configurations at varying flow rates. Figure 2a, b presents the simulation results of the CMC-A. The main flow direction was indicated by solid red arrows, and the positions of the selected cross-sections were marked by black dashed lines with section numbers increasing along the main flow direction. The black solid arrows represented the secondary flow vortex directions. The colored contours with legends 1 and 2 indicated the magnitudes of the main and secondary flow velocities, respectively. These results demonstrated the significant influence of channel geometry, cross-sectional position, and flow rate variations on the flow characteristics. Similarly, the main simulation results of CMC-B were shown in Fig. S1.

At a flow rate of 200 μL·min^−1^, both CMC-A and CMC-B showed enhanced main and secondary flows generated near the obstacle regions. For CMC-A, the secondary flow vortex direction remained consistent near different obstacle positions. The intensity of the secondary flows gradually increased from the initial obstacle region to the final obstacle region (Fig. 2c). For CMC-B, the staggered arrangement of obstacles exhibited distinct behaviors. When only vertical or horizontal obstacles were considered, the secondary flow vortices near the obstacle regions maintained a uniform direction, and the intensity of the secondary flows increased at a relatively slow rate. When vertical and horizontal obstacles were considered simultaneously, the secondary flow vortices exhibited periodic fluctuations in both direction and intensity. The directions of the secondary flow vortices near the two adjacent obstacles were vertical (Fig. S1b). A comparative analysis of the maximal secondary flow velocities near different obstacle regions and flow rates between the two-channel configurations was illustrated in Fig. 2d, e. The secondary flow intensity in CMC-A was significantly higher than that in CMC-B, highlighting a notable synergistic enhancement effect. Previous studies demonstrated that progressively enhanced secondary flows facilitated the co-focusing of multi-sized particles^37^. Therefore, based on the simulation results, CMC-A outperformed CMC-B.

**Fig. 3:**
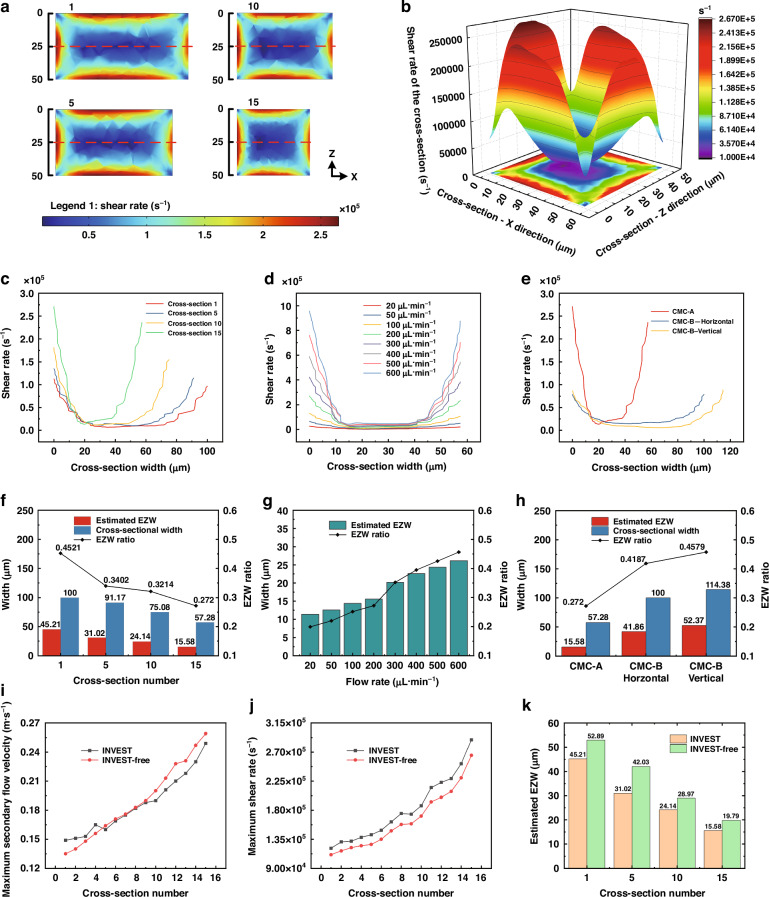
Shear-rate analysis in CMC-A **a** Shear-rate distribution at different cross-sections (flow rate: 200 μL·min^−1^). Legend 1 represents the shear rate ranging from 10,100 to 265,000 1·s^−1^. **b** 3D schematic of the shear-rate distribution at the 15th cross-section, illustrating the variation in the shear gradient (flow rate: 200 μL·min^−1^). The simulation results for the shear rate of CMC-B are shown in Fig. S1. **c** Shear-rate distribution extracted along the red dashed lines in 1st, 5th, 10th, and 15th cross-sections of CMC-A (flow rate: 200 μL·min^−1^). **d** Shear-rate distribution of CMC-A at the 15th cross-section (flow rates: 20–600 μL·min^−1^). **e** Comparison of shear-rate distributions between CMC-A and CMC-B at the 15th cross-section (flow rate: 200 μL·min^−1^). **f** Estimated EZW and its ratio at the four cross-sections of CMC-A (flow rate: 200 μL·min^−1^). **g** Estimated EZW and its ratio at the 15th cross-section of CMC-A (flow rates: 20–600 μL·min^−1^). **h** Comparison of estimated EZWs and their ratios between CMC-A and CMC-B at the 15th cross-section (flow rate: 200 μL·min^−1^). The shear-rate distribution along the horizontal centerline of the CMC-B cross-section was shown in Fig. S3, including plots of shear rate as a function of cross-section position and flow rate, as well as the estimated EZW and their ratios. Comparison of maximum secondary flow (**i**), maximum shear rate (**j**), and estimated EZWs (**k**) at different cross-sections for INVEST and INVEST-free solutions in CMC-A (flow rate: 200 μL·min^−1^). INVEST and INVEST-free represent viscoelastic and non-viscoelastic solutions, respectively

In addition to simulating the main and secondary flows, we also simulated the shear rate of the cross-section to explore the variation in the shear-rate gradient under the INVEST condition and thereby theoretically estimated the equilibrium zone inside the channels. The shear rate induced a normal stress difference, which generated an elastic force. The magnitude of the elastic force was proportional to the variation of the shear rate. Shear-rate distribution simulations at a flow rate of 200 μL·min^−1^ (Figs. 3a and S2) revealed significantly lower values in central and corner regions compared to near-wall areas. Particle movements from equilibrium positions to high-shear regions increased the normal stress differences, generating elastic restoring forces toward equilibrium (Fig. 3b). According to previous studies, particles tend to migrate toward regions characterized by low shear rates or small shear-rate gradients^38^. Therefore, the spatial extent of this low-shear-rate gradient region can serve as an indirect indicator of the equilibrium zone. Specifically, in a rectangular cross-section, the horizontal extent of this region was generally larger than the vertical extent, making the horizontal centerline a more representative measure of the overall low-gradient width. Accordingly, we extracted the shear-rate profile along the red dashed lines shown in Fig. 3a and defined the EZW as the region in which the shear-rate gradient approached zero (|*d*$\dot{\gamma }$/*dx* | < 0.01 s^−1^·μm^−1^) to theoretically evaluate the focusing width of the particles. Additionally, the EZW ratio was defined as the ratio of EZW to the channel width, indicating the relative proportion of the equilibrium zone within a cross-section. A smaller EZW ratio demonstrated a narrower equilibrium zone and a more significant focusing effect.

Numerical simulations (Figs. 3c–h, and S3) revealed that at a flow rate of 200 μL·min^−1^, both CMC-A and CMC-B configurations exhibited increased shear rates with channel constriction. For CMC-A, the estimated EZW and its ratio demonstrated progressive attenuation along the main flow direction. In contrast, CMC-B displayed different trends. Horizontally aligned obstacle arrays induced a stepwise increase in EZW and its ratio, whereas they decreased within vertically aligned obstacle array configuration along the main flow direction. At identical obstacle regions, the shear-rate gradient and estimated EZW of both channel configurations increased with rising flow rate. Comparative analysis revealed that CMC-A exhibited a higher shear-rate gradient and smaller estimated EZW under equivalent conditions. This could generate stronger elastic driving forces, significantly improving the particle migration efficiency.

Furthermore, we conducted simulations using non-viscoelastic solutions in CMC-A at a flow rate of 200 μL·min^−1^ to compare and verify the above results, as shown in Fig. 3i–k. INVEST and INVEST-free represented the simulation results of viscoelastic and non-viscoelastic solutions, respectively. Comparing the results, we concluded that the differences in maximum secondary flow velocity between the two conditions were relatively small, with an average absolute percentage difference of 4.54%. This small difference indicated that the INVEST method exhibited a minimal impact on the secondary flow change. Moreover, the maximum shear rate in the viscoelastic solution showed a significant increase of 10.12% compared with that in the non-viscoelastic solution. This increase in the maximum shear rate reflected a higher shear-rate gradient, which generated a stronger and more concentrated elastic force, leading to a reduction in EZW and thus achieving a more precise focusing. As shown in Fig. 3k, the estimated EZW under the INVEST condition was significantly smaller than that under the INVEST-free condition. As a result, INVEST-based CMC-A outperformed CMC-B in 3D multi-sized particle co-focusing due to its progressively enhanced secondary flow intensity, stable secondary flow vortex direction, and continuously increasing shear-rate gradients.

### Investigation of HA concentrations on particle focusing

We first carried out the focusing experiments to investigate the influence of HA concentrations on particle focusing. We conducted a detailed observation of particle migration from dual views (top and side views) and assessed the particle focusing performance using two quantitative indicators: focusing efficiency and focusing width. We utilized a high-speed camera to capture 12,000 consecutive frames over a 3-s acquisition period, fully recording the spatial distribution of particles in the observation area. The focusing efficiency was defined as the ratio of the number of focused particles to the total number of particles. In addition, we conducted fluorescent stripe imaging experiments and extracted the fluorescence intensity distribution curve using a custom-written MATLAB image-processing program. The focusing width was quantified using the full width at half maximal criterion (Fig. S4). This dual-view (top and side views) and dual-parameter (focusing efficiency and focusing width) evaluation system effectively revealed the multidimensional characterizations of particle focusing dynamics at different flow conditions.

**Fig. 4:**
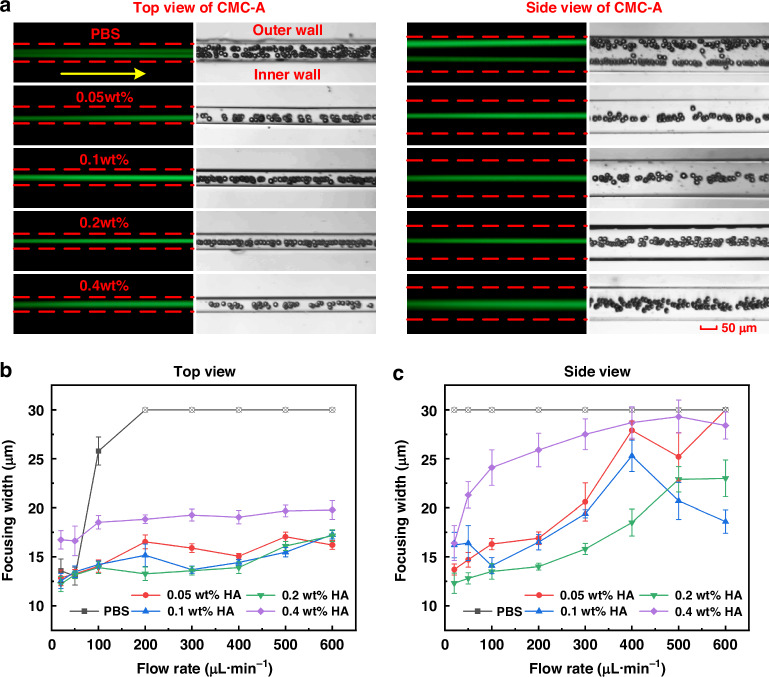
Focusing behavior of 15 μm particles in CMC-A with pure PBS and HA-PBS solutions at varying concentrations (0.05, 0.1, 0.2, and 0.4 wt%) **a** Dual-view (top and side) fluorescent images and high-speed camera images (stacked 100 frames) at a flow rate of 200 μL·min^−1^. The yellow arrows indicate the flow direction, and the red dashed lines represent the channel walls. Scale bar: 50 μm. The complete experimental results were shown in Figs. S5–S9. **b** Focusing width as a function of flow rate (top view). **c** Focusing width as a function of flow rate (side view)

Based on the flow parameters determined by numerical simulation (20–600 μL·min^−1^), this study first explored the regulation mechanism of different concentrations (0.05 wt%, 0.1 wt%, 0.2 wt%, and 0.4 wt%) of HA-PBS solution and pure PBS solution on the focusing behavior of 15 μm particles. Figure 4a showed the fluorescent stripes and high-speed camera images for the five types of solutions at a flow rate of 200 μL·min^−1^ (red dashed lines marking the channel walls and yellow arrows indicating the main flow direction). The experimental results indicated that particles in pure PBS solution exhibited a non-focused feature with double fluorescent stripes, whereas particles in HA-PBS solutions performed single-streamline focusing, verifying the importance of viscoelastic fluids in particle focusing. The complete experimental results were presented in Figs. S5–S9.

We calculated the focusing widths with different HA concentrations and flow rate conditions (Fig. 4b, c). For situations in which particle focusing was not achieved, we used the doubled particle diameter for representation and marked them with a circle containing an ‘×‘ in the graph. From the top view, in HA-PBS solutions with concentrations of 0.05, 0.1, and 0.2 wt%, high-precision focusing (with a focusing width below 1.5 times the particle diameter) was achieved over a broad flow rate range (20–600 μL·min^−1^). From the side view, as the flow rate increased, the focusing width exhibited a significant increase. This phenomenon was attributed to the further enhancement of the secondary flow induced by the increased flow velocity, resulting in fluctuations in the particle equilibrium positions. As the flow rate continued to increase, the particles began to migrate along the direction of the secondary flow vortices, resulting in multiple fluorescent stripes with decreased peak intensity, and thus the focusing widths decreased. Specifically, in three types of HA-PBS solutions (0.05, 0.1, and 0.2 wt%), the ranges of the flow rate for achieving high-precision focusing were 20–300 μL·min^−1^, 20–300 μL·min^−1^, and 20–400 μL·min^−1^, respectively. When the solution concentration increased to 0.4 wt%, the high-precision focusing flow rate range was 20–50 μL·min^−1^, which was significantly narrower than that with lower-concentration solutions. This was mainly attributed to the markedly increased viscosity and the prolonged relaxation time. The elevated viscosity reduced the local shear rate and weakened the inertial effects and secondary flows, which were crucial for maintaining stable particle focusing. Meanwhile, the extended relaxation time increased the Wi and altered the El, resulting in a flow field more dominated by elastic forces and inducing broadened particle trajectories and the onset of elastic instabilities. Nevertheless, these results initially demonstrated that our CMCs induced the inertial migration, viscoelastic effect, and enhanced secondary flow simultaneously, and then systemically formed the INVEST effect for high-precision particle focusing.

In addition, the range of the flow rate for achieving high-precision focusing from the top view was broader than that from the side view, which indicated particle focusing in the horizontal direction (top view) was more efficient than that in the vertical direction (side view). This difference was mainly caused by the gradually increased height of the obstacles and the shrinking of the channel width in the horizontal direction. They led to an increase in the shear-rate gradient along the horizontal direction, making the elastic force acting on the particles stronger and more concentrated, thereby reducing the EZW and enabling high-precision particle focusing at the center of the channel. On the contrary, the obstacles' height and channel depth were identical in the vertical direction. There were no gradually increased elastic and secondary flow forces to stabilize particle trajectories, and thus leading to the fluctuations of the equilibrium positions in the vertical direction (side view).

We also conducted corresponding experiments using CMC-B to compare the focusing behavior between the two-channel configurations. The experimental results and comparisons of the focusing patterns are provided in the Supplementary Materials (Figs. S11–S15). These results indicate that both channel types exhibit similar focusing behavior at lower flow rates. However, as the flow rate increases, CMC-A demonstrates markedly superior focusing performance relative to CMC-B, achieving high-precision particle focusing over a broader range of flow rates than CMC-B. In conclusion, we achieved a dynamic balance among inertial, elastic, and secondary flow drag forces with a wide flow rate range (20–400 μL·min^−1^) in 0.2 wt% HA-PBS solution, resulting in high-precision 3D particle central focusing.

### Investigation of two CMC configurations on particle focusing

**Fig. 5:**
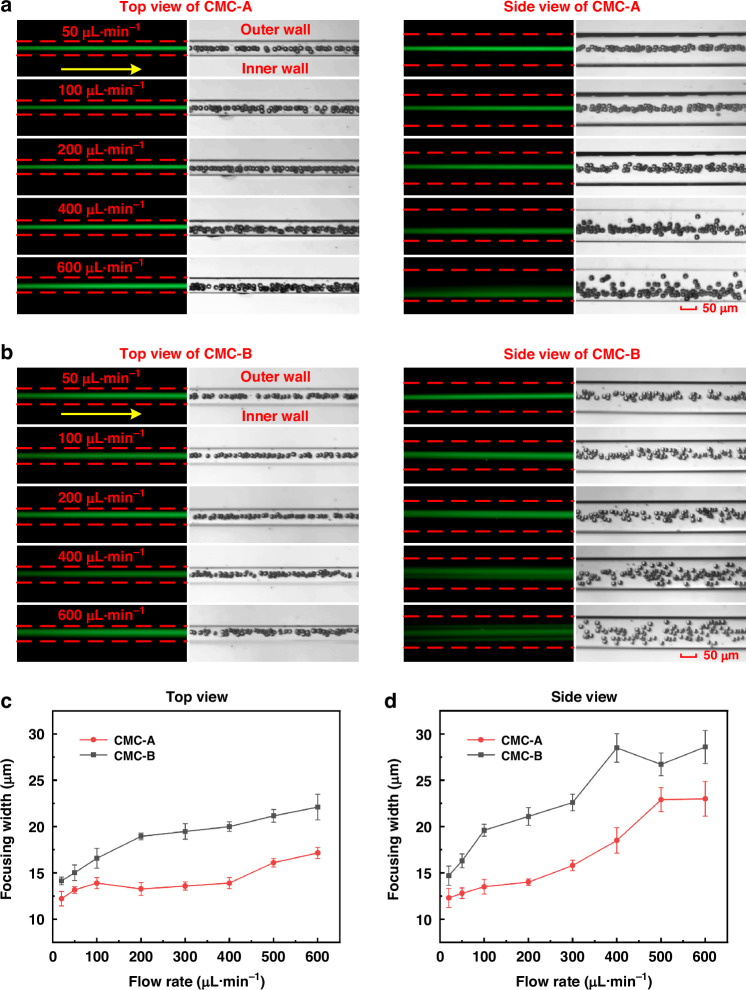
Focusing behavior of 15 μm particles in CMC-A and CMC-B with 0.2 wt% HA-PBS solution **a** Dual-view (top and side) fluorescent images and high-speed camera images (stacked 100 frames) in CMC-A at flow rates ranging from 50 to 600 μL·min^−1^. The yellow arrows indicate the flow direction, and the red dashed lines represent the channel walls. Scale bar: 50 μm. **b** Dual-view (top and side) fluorescent images and high-speed camera images (stacked 100 frames) in CMC-B at flow rates ranging from 50 to 600 μL·min^−1^. The complete experimental results for CMC-A and CMC-B were presented in Figs. S8 and S10. **c** Focusing width in CMC-A and CMC-B as a function of the flow rate (top view). **d** Focusing width in CMC-A and CMC-B as a function of the flow rate (side view)

Fluorescent stripe and high-speed camera images at different flow rates are shown in Fig. 5a, b, respectively. From a top view, the focusing performance of the two-channel configurations exhibited minimal differences. Efficient particle focusing can be achieved at flow rates ranging from 20 to 600 μL·min^−1^. However, from the side view, CMC-A performed significantly superior focusing performance compared to CMC-B, which can be directly observed from the fluorescent stripe and high-speed camera images. High-precision particle focusing could not be realized in CMC-B when the flow rate was larger than 100 μL·min^−1^.

We also calculated the focusing widths of particles in both CMC-A and CMC-B (Fig. 5c, d). The results showed that high-precision 3D focusing can be realized at flow rates ranging from 20–400 μL·min^−1^ in CMC-A, while they were only 20–200 μL·min^−1^ in CMC-B, demonstrating a broader application range and a higher throughput of CMC-A. In addition, the focusing widths in CMC-A were smaller than those in CMC-B at all flow rates. These results indicated that CMC-A was more suitable for achieving high-precision 3D particle focusing than CMC-B.

### Different types of particles focusing in CMC-A

**Fig. 6:**
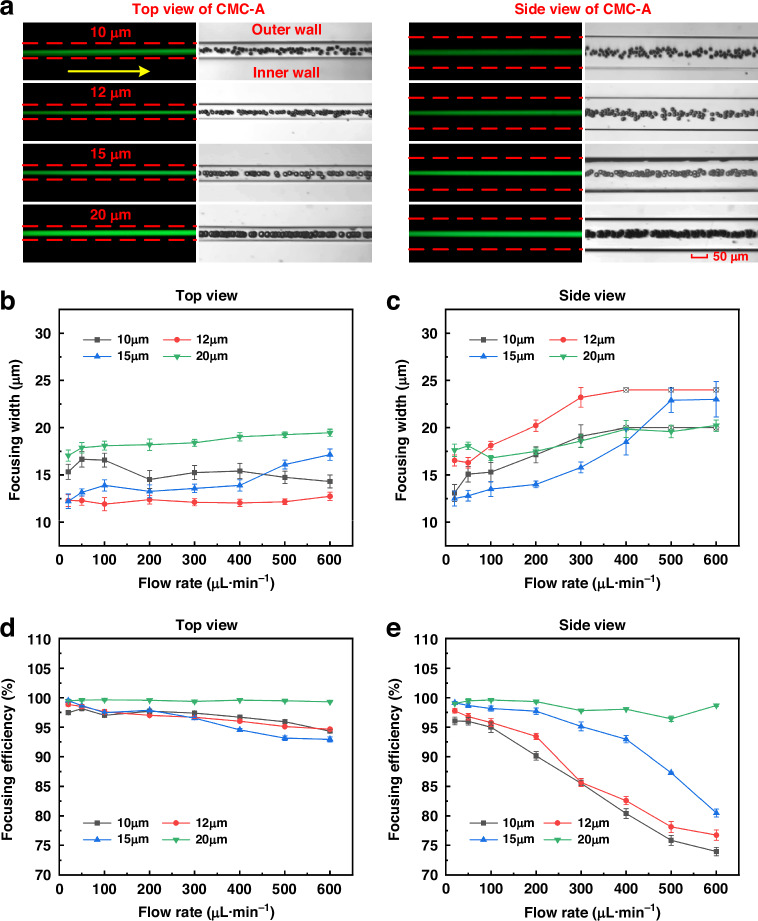
Focusing behavior of particles with different diameters in CMC-A with 0.2 wt% HA-PBS solution **a** Dual-view (top and side) fluorescent images and trajectories (stacked 100 frames) of particles (10, 12, 15, and 20 μm) at a flow rate of 50 μL·min^−1^. The yellow arrows indicate the flow direction, and the red dashed lines represent the channel walls. Scale bar: 50 μm. The complete experimental results were shown in Figs. S16–S18. **b** Focusing width as a function of the flow rate (top view). **c** Focusing width as a function of the flow rate (side view). **d** Focusing efficiency as a function of the flow rate (top view). **e** Focusing efficiency as a function of the flow rate (side view)

We further conducted experiments using fluorescent particles with different diameters (10, 12, 15, and 20 μm). The results of single-sized particle focusing were shown in Figs. 6a and S16–S18. From the top view, the images of the fluorescent stripes and high-speed camera images demonstrated that all four types of particles can be efficiently focused at flow rates of 20–600 μL·min^−1^. However, from the side view, the emergence of dual-focused particle streams became evident, and smaller particles exhibited greater susceptibility to flow rate variations. For instance, 10 μm particles can be efficiently focused to a single streamline at a flow rate below 200 μL·min^−1^. When the flow rate was larger than 200 μL·min^−1^, more than one equilibrium position appeared (see fluorescent images in Fig. S16). However, for 20 μm particles, single-streamline focusing can be realized even at 600 μL·min^−1^. This phenomenon could be attributed to the fact that the inertia lift forces acting on a particle were closely related to its size. Small particles with weak inertial lift forces were more susceptible to Brownian motion or fluid disturbances induced by secondary flow than large particles. Therefore, single-streamline focusing of large particles was easier to accomplish, and the focusing pattern was more stable than that of smaller particles.

In order to further verify the above-mentioned view, we also calculated the focusing width and efficiency for four types of particles from dual views (Fig. 6b–e). Larger particles performed a smaller focusing width to diameter ratio and a higher focusing efficiency than smaller particles, demonstrating a more stable single-streamline focusing. In addition, it was obvious that the focusing width increased and the focusing efficiency decreased with the increase in flow rate in the side view. The underlying reason has been interpreted before. No additional elastic force and secondary flow drag force were generated due to the identical channel depth and obstacles height in the vertical direction (side view). Nevertheless, we achieved high-precision and high-efficiency 3D focusing for four particle types at a broad flow rate range. For 10 μm and 12 μm particles, high-precision 3D focusing was accomplished at flow rates in a range from 20 to 100 μL·min^−1^ with maximal efficiencies of 96.05% and 97.76%, respectively, while the flow rate ranges were 20–400 μL·min^−1^ and 20–600 μL·min^−1^, and the maximal efficiencies were 99.14% and 99.61% for 15 μm and 20 μm particles, respectively. Moreover, particles were focused on the centerline of the channel, which makes it more suitable for single-cell analysis, especially for flow cytometry. All the results demonstrated that our CMC-A provided an efficient way for high-precision and high-efficiency 3D particle central focusing.

### Multi-sized particle co-focusing in CMC-A

**Fig. 7:**
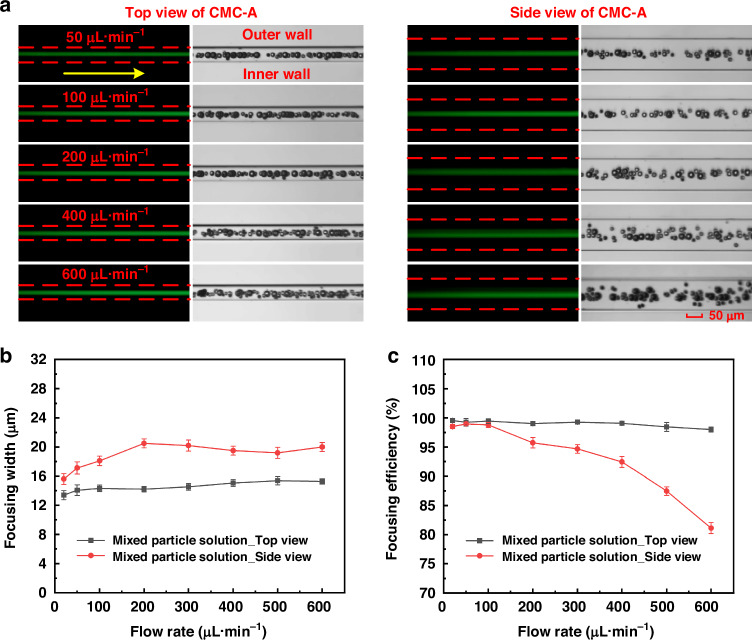
Focusing behavior of particle mixtures (10, 12, 15, and 20 μm; 1:1:1:1 ratio) in CMC-A with 0.2 wt% HA-PBS solution **a** Dual-view (top and side) fluorescent images and high-speed camera images (stacked 100 frames) at flow rates ranging from 50 to 600 μL·min^−1^. The yellow arrows indicate the flow direction, and the red dashed lines represent the channel walls. Scale bar: 50 μm. The complete experimental results were shown in Fig. S19. **b** Focusing width as a function of the flow rate. **c** Focusing efficiency as a function of the flow rate

To verify the multi-sized particle co-focusing performance, we carried out co-focusing experiments using a mixed particle suspension with 10, 12, 15, and 20 μm particles (in a 1:1:1:1 ratio). Fluorescent stripes and high-speed camera images (Figs. 7a and S19, Videos S1 and S2) showed that multi-sized particles performed a high-precision 3D co-focusing in the observation area within a flow rate range of 20–300 μL·min^−1^. Beyond the observation region, the channel exhibits a sudden expansion in cross-section, which disrupts the established focusing pattern and leads to particle divergence. When the flow rate was larger than 300 μL·min^−1^, high-precision co-focusing was obtained in the top view, while co-focusing in the side view was disturbed. The underlying reason was that the channel width gradually shrank from inlet to outlet, and the obstacles on the inner wall changed accordingly, resulting in an increase in secondary flow and shear rate. The enhanced secondary flow drag force and elastic force improved the efficiency of adjusting particle position, especially for small particles. However, the channel depth and the obstacle height on the top wall were constant, leading to identical secondary flow drag force and elastic force, which could not be used for modifying different size particle position.

In addition, large particles (15 and 20 μm) were concentrated at the main equilibrium position in the center of the channel, whereas small particles (10 and 12 μm) offset to the vicinity of the main equilibrium position, especially when the flow rate was greater than 300 μL·min^−1^ (see high-speed camera images). The different particle focusing behaviors could be attributed to the competing interactions of the forces. For large particles, the inertial lift and elastic forces were at a comparable magnitude level. They jointly dominated the focusing pattern, significantly surpassing the effects of secondary flow, thereby maintaining the stable equilibrium at the channel centerline. In contrast, small particles were more sensitive to the secondary flow effects. After reaching their primary equilibrium positions, they remained subject to persistent perturbation. These perturbations drove partial small particles migrating away from the central region. Moreover, as the flow rate increased, a shear-thinning effect occurred in the low-concentration polymer solution, where the viscosity decreased due to the increased flow rate. This resulted in a slower growth rate of the elastic forces, whereas the inertial lift acting on the particles increased more rapidly. Particle focusing gradually transferred to being dominated by the inertial lift force. Meanwhile, the impact of the secondary flow on particle focusing was strengthened, causing the particles (especially smaller particles) to move along the secondary flow vortex. Therefore, 3D co-focusing of multi-sized particles can hardly be achieved at a relatively large flow rate.

To quantitatively analyze the results, the focusing width and efficiency varying with flow rate were calculated and compared (Fig. 7b, c). We found that the maximal focusing width was only 20.5 μm, indicating a high-precision 3D co-focusing within a broad flow rate range (20–600 μL·min^−1^). Moreover, the focusing width decreased with the increasing flow rate (>200 μL·min^−1^). It was different from the single-sized particle focusing results, in that the focusing width increased with the increasing flow rate. This did not indicate a better focusing performance. As analyzed before, the focusing pattern of a few small particles was disturbed due to the increasing secondary flow. These small particles exhibited weaker fluorescence than large particles, which was easily neglected by the camera with identical exposure. Therefore, the corresponding fluorescent stripes were thinner than they should be, leading to a small focusing width. In addition to the focusing width, the focusing efficiency gradually decreased as the flow rate increased, but it remained above 95% within a flow rate range below 300 μL·min^−1^. These results provided sufficient experimental evidence that our INVEST- and CMC-based focusing chips performed an excellent 3D central co-focusing of multi-sized particles in both high-precision and high-efficiency manners.

In conclusion, the experimental results were highly consistent with the theoretical simulation results. High-speed camera images revealed particle focusing at the channel center, which was consistent with the estimated equilibrium zone in the simulations. The secondary flow induced by inertial effects in the CMC-A device suppressed the corner attraction effect of viscoelastic migration. The viscoelastic effect accelerated the balancing process of particles in the channel, further reducing the deviation of the particle equilibrium positions. Meanwhile, the variable and enhanced secondary flow played a key role in achieving multi-sized particle 3D co-focusing. Compared to single viscoelastic or inertial focusing, the INVEST-based particle focusing method provided a more efficient and precise way for 3D co-focusing of multi-sized particles.

### WBC focusing in CMC-A

**Fig. 8:**
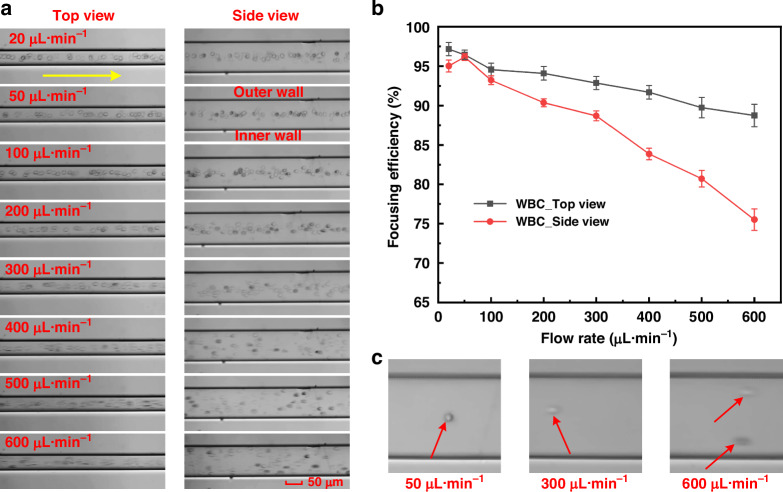
Experimental results of WBCs focusing in CMC-A with varying flow rates **a** High-speed images (stacked 50 frames) showing the location of WBCs captured in the observation area at flow rates ranging from 20 to 600 μL·min^−1^. The yellow arrow indicates the fluid flow direction, and the red dashed lines represent the channel walls. Scale bar: 50 μm. **b** Focusing efficiency of WBCs as a function of flow rate. The maximal focusing efficiency is 96.14% at a flow rate of 50 μL·min^−1^. **c** Morphology of WBCs at flow rates of 50, 300, and 600 μL·min^−1^

White blood cells focusing is crucial in flow cytometry, significantly influencing the accuracy and reliability of cell counting and analysis. Here, we carried out the focusing experiments using WBCs to further evaluate the cell focusing performance of our INVEST-based method. WBCs consisted of different subpopulations with diameters ranging from 7 μm to 20 μm, which can be an ideal model for verifying the 3D co-focusing of our CMCs. The cell suspensions were infused into CMC-A at different flow rates (20–600 μL·min^−1^). Figure 8 showed the cell position images in the observation area (the complete experimental results were shown in Videos S3 and S4).

In the top view, the WBCs were focused to a narrow single streamline at flow rates ranging from 20 to 300 μL·min^−1^. When the flow rate exceeded 300 μL·min^−1^, the WBCs exhibited significant disturbance, and their positions deviated from the channel center. From the side view, however, the co-focusing of WBCs could only be obtained at flow rates ranging from 20 to 200 μL·min^−1^. When the flow rate exceeded 200 μL·min^−1^, some cells began to migrate toward the channel walls. As the flow rate further increased, the trend of WBCs accumulation on channel walls emerged. The underlying reasons mainly included two aspects. On one hand, as discussed above, the different configurations of the obstacles in horizontal and vertical directions can interpret this phenomenon. On the other hand, cell deformation could induce different focusing behaviors. At low flow rates, the inertial lift force and elastic force were balanced, and the secondary flow drag force was moderate. The morphology of the WBCs remained approximately spherical, thereby forming a stable dynamic equilibrium system. With an increase in flow rate, the viscoelastic fluid underwent shear thinning, the growth rate of the elastic force slowed, and the inertial lift force gradually dominated the cell migration process. The difference in the growth rates of the two forces disrupted the original balance, causing the cells to be stretched or compressed, and their volume decreased (gradually changing from a nearly spherical shape to a flat ellipsoid or even strip-like morphology, Fig. 8c). As illustrated in Eqs. (2), (4), and (6) (*F*_L_ ~ *a*⁴, *F*_E_ ~ *a*³, and *F*_D_ ~ *a*). The equivalent radius of the deformed cells decreased, resulting in a reduction of the three forces. However, the rate of decline of *F*_D_ was significantly lower than that of *F*_L_ and *F*_E_. Therefore, the influence of the secondary flow drag force on the cells was significantly enhanced, causing the cells to move along with the vortex and thus disturbing the stable focusing patterns.

In addition, we calculated the focusing efficiencies at flow rates ranging from 20 to 600 μL·min^−1^. The results revealed that the WBCs focusing efficiency exceeded 90% at a flow rate range from 20 to 400 μL·min^−1^ in the top view, while in the side view, they were only 20–200 μL·min^−1^. Moreover, we found that the focusing efficiency of WBCs was lower than that of co-focusing of multi-sized particles. The size and nonuniformity of particles and cells can explain the difference in focusing efficiency. The commercial particles usually had a uniform size with a much smaller coefficient of variation than cells. We used the particle mixtures with a size distribution in a range from 10 μm to 20 μm, while the WBCs usually had diameters ranging from 7 μm to 20 μm. Some monocytes were even greater than 20 μm. The size distribution of WBCs was larger than the particle mixture. Moreover, WBCs could deform when suffering from a high flow rate, which also influenced the focusing performance. Nevertheless, we achieved a highest 3D focusing efficiency of 96.14% for WBCs at a flow rate of 50 μL·min^−1^, demonstrating the capability of our INVEST- and CMC-based particle focusing method for achieving high-precision 3D co-focusing of WBCs.

## Discussion

This study presented a 3D composite microchannel, generating and combining inertial effect, viscoelastic effect, and enhanced secondary flow, forming the INVEST, for high-precision 3D co-focusing of multi-sized particles. Theoretical simulations demonstrated that the composite microchannel with an in-phase obstacle configuration (CMC-A) generated enhanced secondary flows with consistent vortex direction and progressive intensity, coupled with the gradually increased inertial migration and shear-rate gradients, thereby establishing a dynamic force equilibrium among 10–20 μm particles. Compared with the out-of-phase obstacle configuration microchannel (CMC-B), CMC-A performed superiorly in suppressing corner attraction effects and maintaining narrow focusing widths, providing a foundation for high-precision and high-efficiency 3D co-focusing applications. We proposed a new metric of equilibrium zone width to assess focusing performance, with simulations indicating a minimal EZW of 15.58 μm. Comprehensive experiments were carried out to verify the presented INVEST and CMC. We achieved stable 3D co-focusing of multi-sized particle mixtures over a broad flow rate range (20–300 μL·min^−1^), with focusing efficiencies of >95% and a maximal co-focusing width of 20.5 μm. Additionally, WBCs focusing experiments with a maximal efficiency of 96.14% further validated the biocompatibility of the system.

The presented INVEST- and CMC-based microfluidic focusing method addresses the fundamental limitations of inertial (size-dependent equilibrium positions) and viscoelastic (corner attraction and secondary flow disruptions) focusing and enables the simultaneous 3D co-focusing of multi-sized particle mixtures and WBCs, offering a functional advantage over other viscoelastic or inertial focusing methods that cannot align heterogeneous populations to a single 3D streamline. Our INVEST-based focusing method could provide a unique and powerful platform for downstream cell analysis applications such as cell cytometry, label-free cell assays, and high-throughput sorting.

## Materials and methods

### Chip design and fabrication

The 3D composite microchannels (CMCs) were designed (Fig. 2a) based on our previous study. Briefly, the outer wall of the main channel was a semicircular arc with a curvature radius of 4200 μm (*R*₁), whereas the inner wall consisted of a spiral line (0.5 turns) with radii ranging from 4000 μm (*R*₃) at the starting point to 4100 μm (*R*₅) at the endpoint. In the horizontal direction, 15 semicircular protruding obstacles were smoothly connected to the inner wall through 100 μm (*r*₃) and 1000 μm (*r*₄) chamfers, with a spacing of 11° (α) between adjacent obstacles. The height of each obstacle was jointly determined by a spiral line (ranging from 4100 μm (*R*₂) at the starting point to 4150 μm (*R*₄) at the endpoint) and the inner wall of the channel. Therefore, the height of the obstacles decreased linearly from 100 μm at the inlet to 50 μm at the outlet, forming a gradient array of obstacles. In the vertical direction, the overall channel depth was constant at 100 μm (*H*₁) with 15 protruding obstacles (50 μm deep, *H*₂) connected through 150 μm (*r*₁) and 80 μm (*r*₂) chamfers. We also designed a straight channel with dimensions of 50 × 100 μm^2^ before the outlet for dual-view particle observation and detection (top and side view). To investigate the impact of the obstacle configuration on particle focusing, the CMCs were designed with two configurations: CMC-A (in-phase configuration: horizontal and vertical obstacles were spatially aligned) (Fig. S20) and CMC-B (out-of-phase configuration: horizontal obstacles were offset by 1/2 period relative to the vertical obstacles) (Fig. S21). Besides, 14 equidistant cylindrical micropillars were integrated at the inlet to prevent clogging. Through the synergistic effects of spiral curvature, obstacle height gradients, and horizontal-vertical obstacle coordination, our design could accomplish a 3D coupling of the inertial effect, viscoelastic effect, and secondary flows, forming INVEST and providing an innovative solution for the high-precision 3D co-focusing of multi-sized particles.

The CMC chips were fabricated via a two-photon 3D printing technique to create an IP-S mold on a glass substrate. We then produced an epoxy resin mold using a replication process to cast polydimethylsiloxane (PDMS) chips. Compared to the directly printed IP-S mold, the epoxy resin mold exhibited superior mechanical strength and reliability, enabling multiple PDMS chip replications without structural failure. Subsequently, the PDMS base was mixed with a curing agent (Sylgard 184, Dow Corning, USA) at a 10:1 ratio by weight. After degassing, the mixture was poured into the epoxy resin mold and cured in an oven at 80 °C for 2 h. The PDMS layer was peeled off, and inlet/outlet holes were punched. Finally, the PDMS layer was cut into individual units and bonded to oxygen plasma-treated glass slides (TS-PL05, TONSON, China). The assembled chip was further annealed at 80 °C for 1 h to enhance the bonding strength. The fabricated chips were presented in Fig. S22.

### Simulation

This study utilized COMSOL Multiphysics (Burlington, MA, USA) to construct a 3D microchannel flow model. The model simulated the flow field distribution under finite Reynolds number conditions by solving the laminar incompressible flow equations. The primary goal was to reveal the spatial distribution of inertial lift force, elastic lift force, and secondary flows inside the CMCs, and to estimate the particle equilibrium positions. To simulate the rheological properties of the 0.2 wt% HA-phosphate buffer saline (PBS) solution, the fluid density and dynamic viscosity were set to 1009.9 kg/m³ and 0.01 Pa·s, respectively^39–44^. The computational domain was discretized using a free tetrahedral mesh with a maximum element size of 10.2 μm. Local grid refinement was performed in critical regions, achieving a minimum element size of 1.33 μm to ensure mesh independence and computational accuracy of the simulation. Finally, the model was validated using experimental data to confirm its reliability.

### Sample preparation

Four types of polystyrene particles (Tianjin Bessler, China) with diameters of 10, 12, 15, and 20 μm were used to characterize the focusing performance of the CMCs. Additionally, four different concentrations (0.05 wt%, 0.1 wt%, 0.2 wt%, and 0.4 wt%) of HA (*Mw* = 1.0 MDa~1.8 MDa, Sigma-Aldrich, USA)-PBS (Sigma-Aldrich, Japan) solutions and pure PBS solution were prepared. These solutions were used to prepare particle suspensions for the focusing experiments. The concentration of the single-sized particle suspension was 5 × 10^5^ particles·mL^−1^. The concentration of the particle mixture, containing 10, 12, 15, and 20 μm particles in a 1:1:1:1 ratio, was also 5 × 10^5^ particles·mL^−1^. The particle specific mass was 1.05 × 10^3^ kg·m^−3^. Each particle contains a mixture of fluorophores that can be excited at a wavelength of 480 nm.

Blood samples were provided by healthy adults from the Xi’an International Medical Center Hospital. All experiments were conducted in accordance with the relevant laws and institutional guidelines in China, and were approved by the Ethics Committee of Northwestern Polytechnical University. To verify the focusing capability of the CMCs for WBCs, 1 mL of whole blood was mixed with 5 mL of red blood cell lysis buffer (E8061, Adamas-Life, China) and incubated at room temperature for 12 min to lyse the red blood cells. The sample was then centrifuged to retrieve the WBCs, and the supernatant was discarded. The WBCs were then mixed with HA-PBS solutions to a final volume of 10 ml with a concentration of approximately (4 ~ 10) × 10⁵ cells·mL^−1^, calculated by a hemocytometer.

### Experimental setup

Focusing experiments were conducted using an inverted fluorescent microscope with a mercury lamp. The filter set included an excitation filter of D480/30×, a dichroic mirror of 505DCLP, and a fluorescent emission filter of D535/40 m, which effectively captured the green fluorescence of the particles excited by a mercury lamp. The particle suspensions were infused into the CMCs using a syringe pump (TYD01-01, LEADFLUID, China), and the flow rates were set from 20 to 600 μL·min^−1^ based on the simulation results. Under dark-field conditions, a CMOS camera (Moticam S5, MOTIC, China) with the corresponding software (Motic Images Plus 3.0, MOTIC, China) was used to capture fluorescent stripe images. Under bright-field conditions, a high-speed camera (M120M, Fu Huang Agile Device, China) and associated software (Revealer Control Center, China) were used to record the experimental images. The high-speed camera recorded the position of the particles in the observation area with an exposure time of 3 μs, a frame rate of 4000 fps, and an image resolution of 640 × 480 pixels. The captured images were processed and analyzed using MATLAB (Mathworks, MA, USA) and ImageJ (NIH, MD, USA) software.

### Ethics declarations

This study was approved by the Medical and Laboratory Animal Ethics Committee of Northwestern Polytechnical University (No. 202502055). All study methods were carried out in accordance with relevant guidelines and regulations. Verbal consent from participants was obtained during the data collection process, as approved by the Medical and Laboratory Animal Ethics Committee of Northwestern Polytechnical University.

## Supplementary information

Supplementary materials

The top-view video of multi-sized particle co-focusing

The top-view video of multi-sized particle co-focusing

The top-view video of multi-sized particle co-focusing

The side-view video of white blood cells co-focusing
