# Supplementary material for: Coupling inertial, viscoelastic, and enhanced secondary flow in a composite microchannel: achieving high-precision multi-sized particle 3D central co-focusing
Source: Microsyst Nanoeng. 2026 Apr 15;12:134. doi: 10.1038/s41378-026-01254-9 (PMC13083988; doi:10.1038/s41378-026-01254-9)
Supplement: Supplementary file 1 — Supplementary materials [file 41378_2026_1254_MOESM1_ESM.docx]

**Supplementary materials**

**Coupling Inertial, Viscoelastic, and Enhanced Secondary Flow in a Composite Microchannel: Achieving High-Precision Multi-Sized Particle 3D Central Co-Focusing**

Tianwei Zhao^1^, Peng Zeng^1^, Chenchen Ji^1^, Xu Yin^2^, Jinxia Li^3^, Xing Chen^1^, Yuanming Ma^1^, Gaobin Xu^1^*, Xichen Yuan^2^*, and Jianguo Feng^1^^,4^*

^1^ School of Microelectronics, Hefei University of Technology, Hefei, Anhui 230601, China.

^2^ School of Mechanical Engineering, Northwestern Polytechnical University, Xi'an, Shaanxi 710072, China.

^3^ Department of Medical Laboratory, Xi'an International Medical Centre Hospital, Xi'an, Shaanxi 710100, China.

^4^ Department of Microsystems Engineering, University of Freiburg, Georges-Koehler-Allee 103, Freiburg 79110, Germany.

*Corresponding authors

Jianguo Feng: [fengjg@hfut.edu.cn](mailto:fengjg@hfut.edu.cn)

Xichen Yuan: [xichen.yuan@nwpu.edu.cn](mailto:xichen.yuan@nwpu.edu.cn)

Gaobin Xu: [gbxu@hfut.edu.cn](mailto:gbxu@hfut.edu.cn)

Table S1 Calculated hydraulic diameter (*D*_h_) and Elasticity number (El) for a 0.2 wt% HA–PBS solution at various channel widths (w = 50–200 μm) with channel height (*h* = 50 μm), fluid density (*ρ* = 1009.9 kg/m³), viscosity (*μ* = 0.01 Pa·s), and relaxation time (*λ* = 0.001 s)^1^.

| *w* (μm) | *D*_h_ (μm) | El (λ=0.001s) |
| --- | --- | --- |
| 50 | 50.00 | 7.92 |
| 75 | 60.00 | 5.50 |
| 100 | 66.67 | 4.46 |
| 125 | 71.43 | 3.38 |
| 150 | 75.00 | 3.52 |
| 175 | 77.78 | 3.27 |
| 200 | 80.00 | 3.09 |


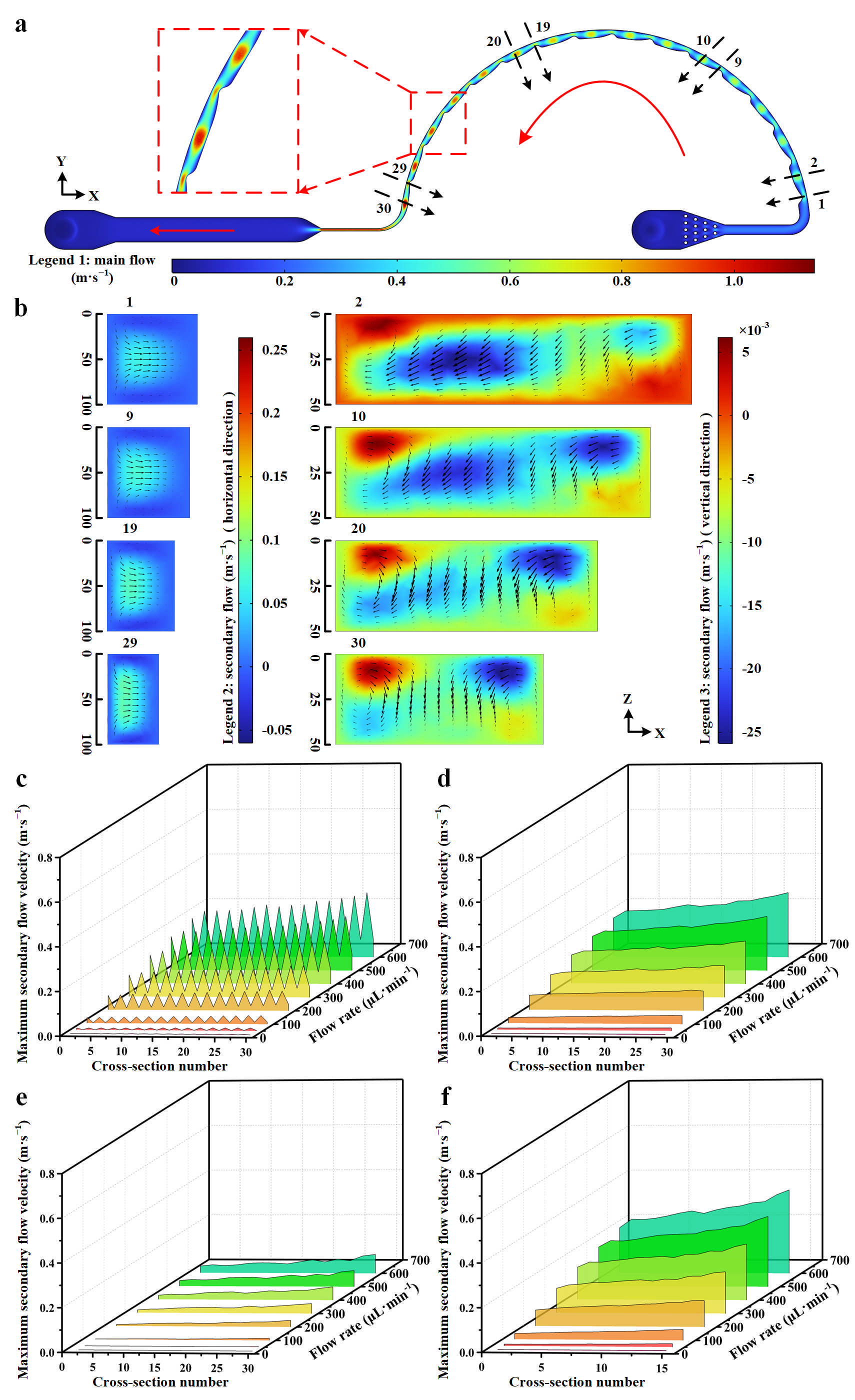


Fig. S1 Theoretical simulation results of CMC-B including the main flow, secondary flow at different cross-sections, and maximal secondary flow velocity as a function of the flow rate. (a) Color contour of the main flow velocity in CMC-B. The red arrow represents the main flow direction, and the dashed black arrows mark the selected cross-sections. Legend 1 shows the main flow velocity ranging from 0 to 1.17 m·s⁻¹. (b) Secondary flows velocity in the four selected cross-sections of CMC-B, including near horizontal and vertical obstacle regions. The secondary flow vortices are indicated by black arrows. Legends 2 and 3 show the secondary flow velocities, ranging from -0.0541 to 0.259 m·s⁻¹ and -0.0263 to 0.0062 m·s⁻¹, respectively. (c) Maximal secondary flow velocity as a function of 30 cross-sections (15 horizontal and 15 vertical obstacles) and flow rate. The flow rates vary from 20 to 600 μL·min⁻¹. (d) Maximal secondary flow velocity as a function of 15 cross-sections (horizontal obstacles) and flow rate. The flow rates vary from 20 to 600 μL·min⁻¹. (e) Maximal secondary flow velocity as a function of 15 cross-sections (vertical obstacles) and flow rate. The flow rates vary from 20 to 600 μL·min⁻¹. (f) The sum of the maximal secondary flow velocities (vertical and horizontal obstacles) as a function of the cross-sections and the flow rate. The flow rates vary from 20 to 600 μL·min⁻¹.


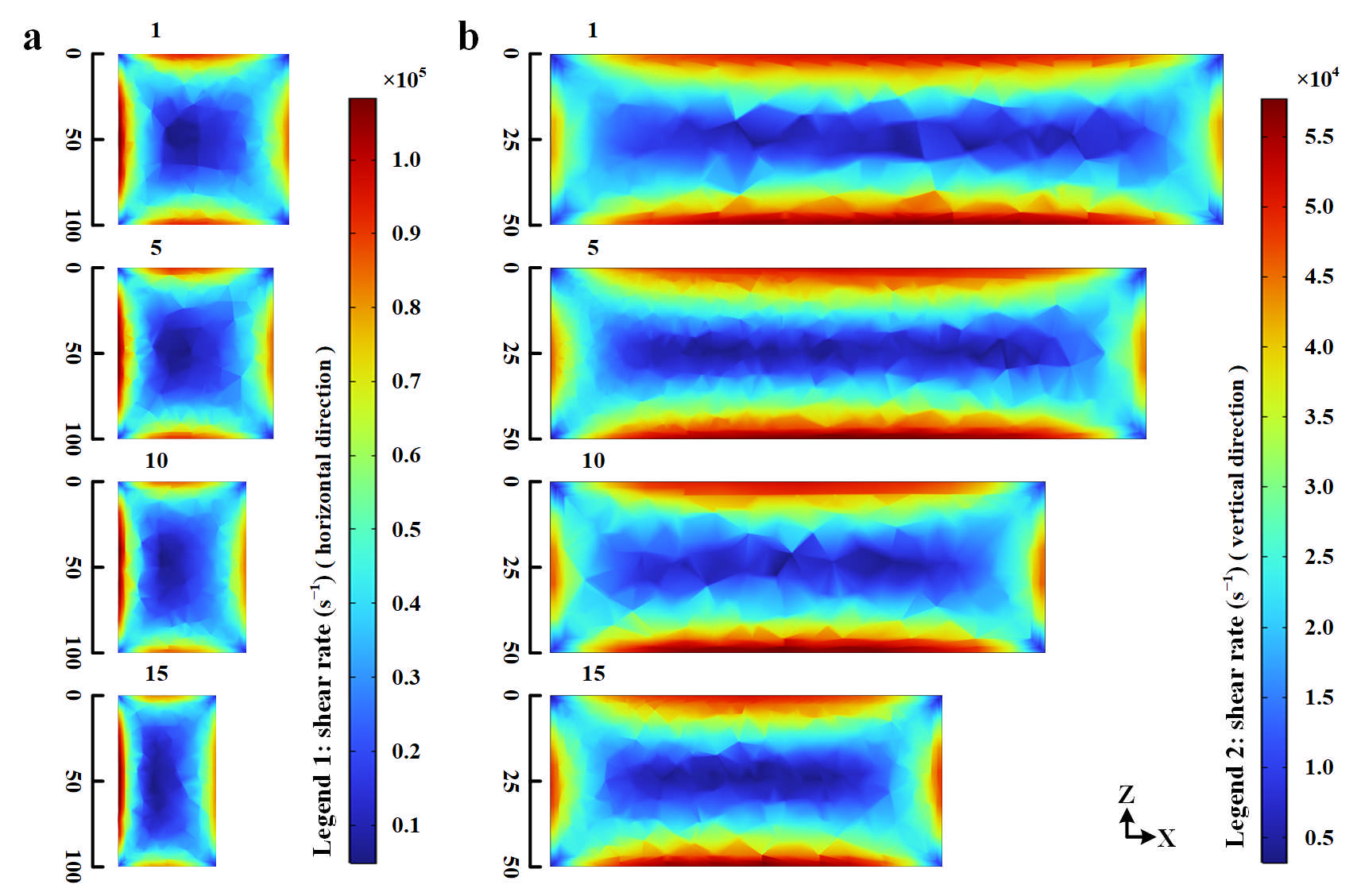


Fig. S2 Simulation results on shear rate of CMC-B. (a) Shear rate distribution of the cross-sections near horizontal obstacle regions. Legend 1 represents the shear rate ranging from 4850 to 108000 1·s⁻¹. (b) Shear rate distribution of the cross-sections near vertical obstacle regions. Legend 2 represents the shear rate ranging from 3210 to 57700 1·s⁻¹.


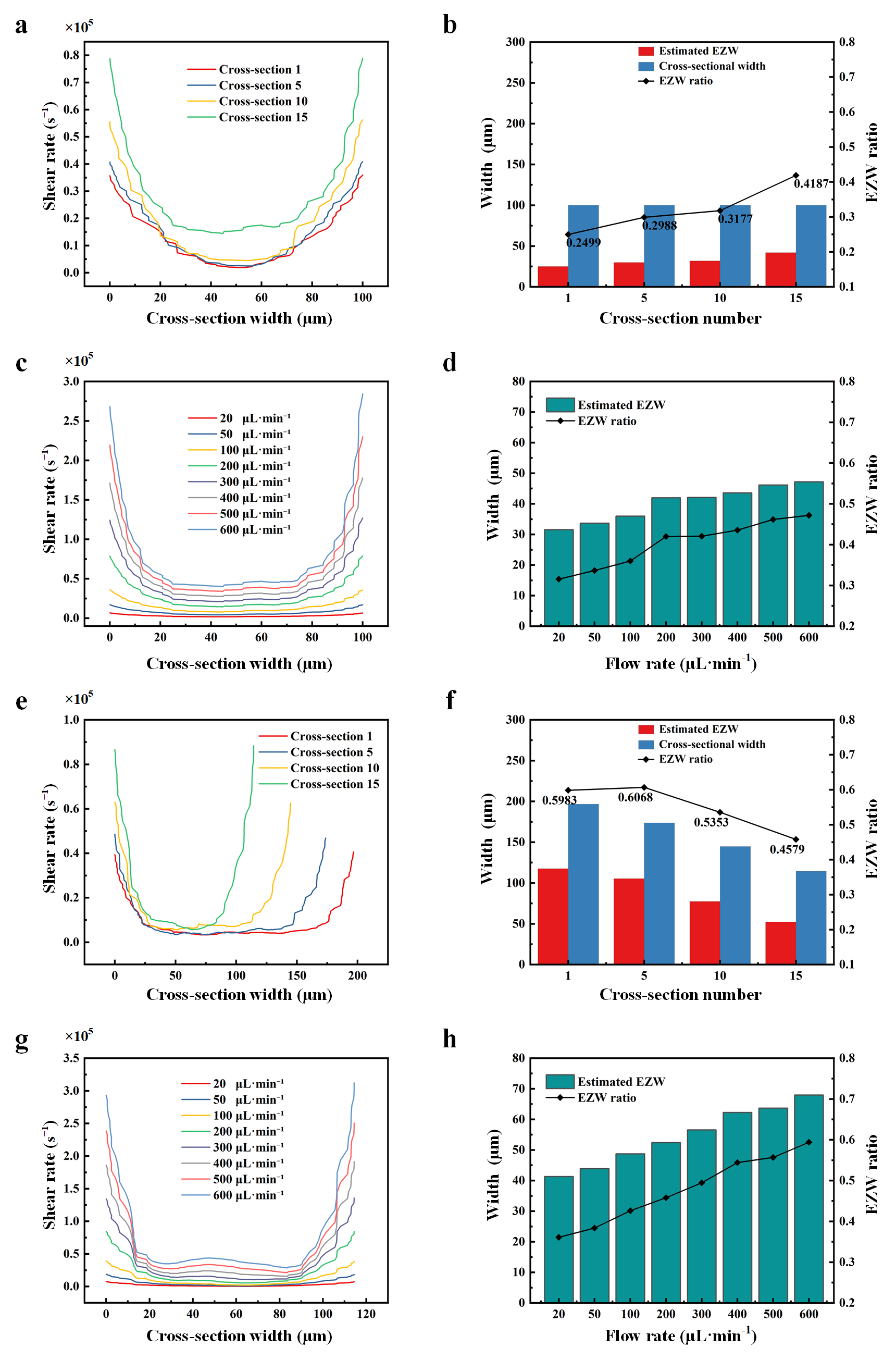


Fig. S3 Shear rate profiles along the short-axis centerline of cross-sections in CMC-B. (a) Shear rate distribution at the 1st, 5th, 10th, and 15th cross-sections of horizontal obstacles (flow rate: 200 μL·min⁻¹). (b) Estimated EZW and its ratio at the 1st, 5th, 10th, and 15th cross-sections of horizontal obstacles (flow rate: 200 μL·min⁻¹). (c) Shear rate distribution at the 15th cross-section of horizontal obstacles at varying flow rates (20–600 μL·min⁻¹). (d) Estimated EZW and its ratio at the 15th cross-section of horizontal obstacles at varying flow rates (20-600 μL·min⁻¹). (e) Shear rate distribution at the 1st, 5th, 10th, and 15th cross-sections of vertical obstacles (flow rate: 200 μL·min⁻¹). (f) Estimated EZW and its ratio at 1st, 5th, 10th, and 15th cross-sections of vertical obstacles (flow rate: 200 μL·min⁻¹). (g) Shear rate distribution at the 15th cross-section of vertical obstacles at varying flow rates (20–600 μL·min⁻¹). (h) Estimated EZW and its ratio at the 15th cross-section of vertical obstacles at varying flow rates (20–600 μL·min⁻¹).


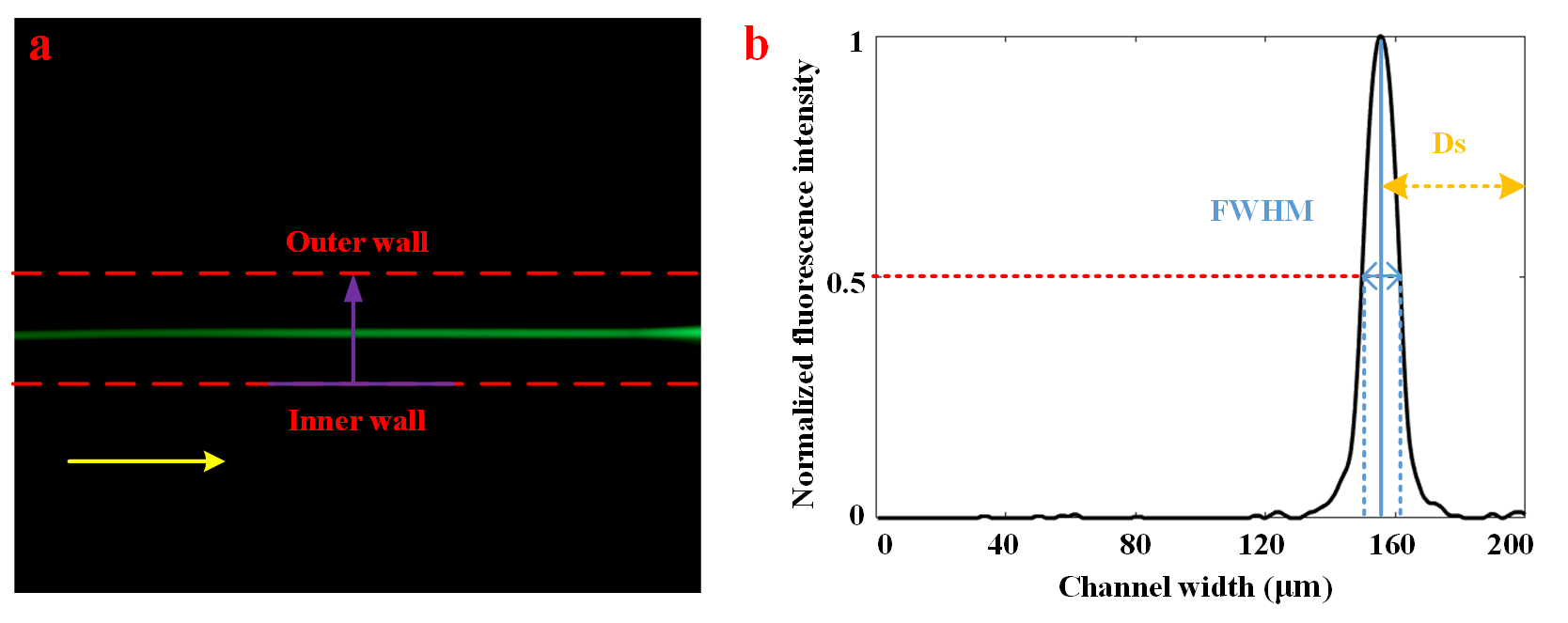


Fig. S4 (a) Fluorescent streak of particle focusing. The yellow arrows show the fluid flow direction, the red dashed lines represent the channel walls, and the purple arrows indicate the position and direction of the image processing. (b) Illustration of the full width at half maximum (FWHM) for the determination of the focusing width.


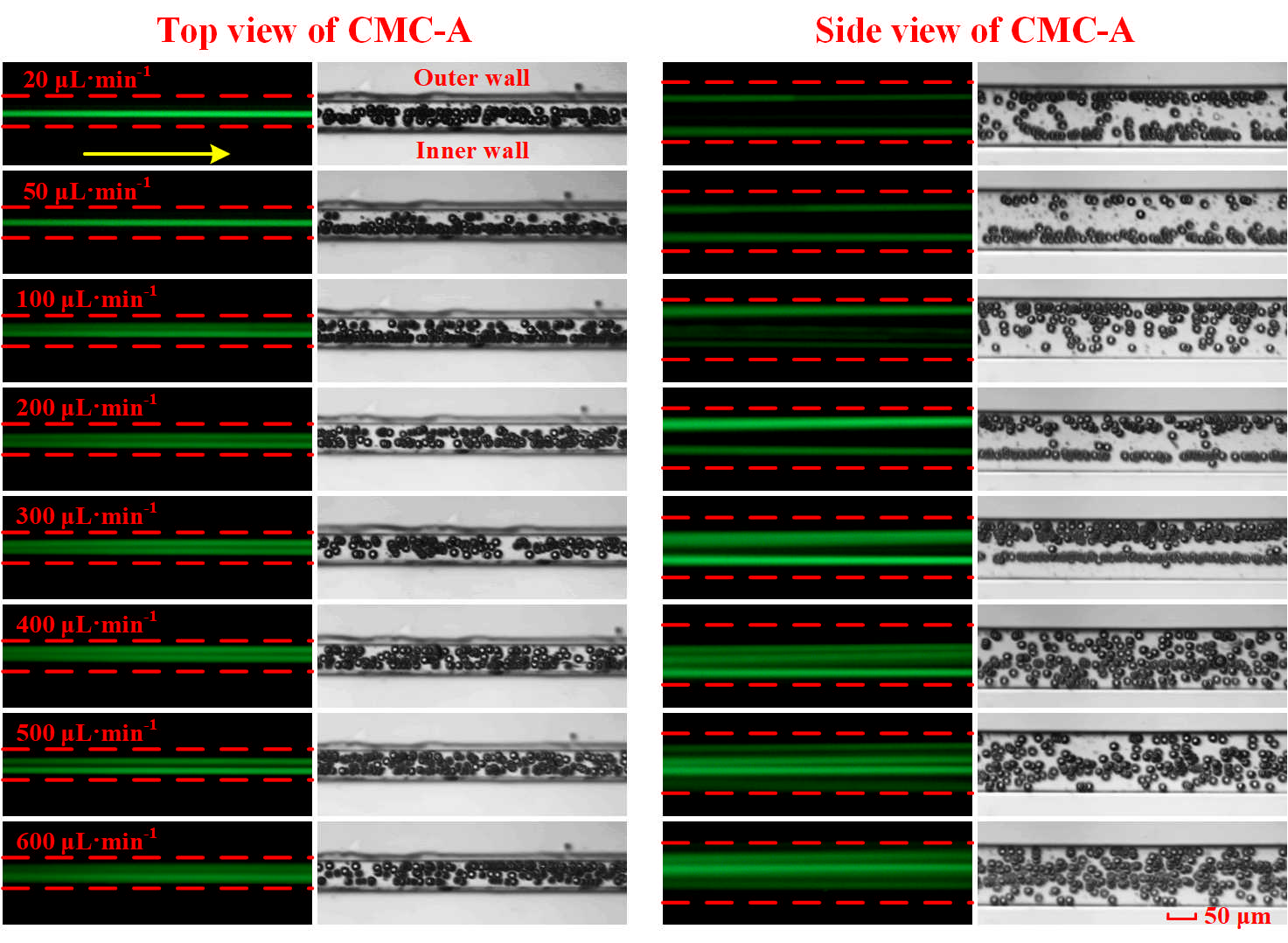


Fig. S5 Focusing behavior of 15 μm particles in CMC-A with pure PBS solution. Dual-view (top and side) fluorescent images and high-speed camera images (stacked 100 frames) at flow rates ranging from 20 to 600 μL·min⁻¹. The yellow arrows indicate the flow direction and the red dashed lines represent the channel walls. Scale bar: 50 μm.


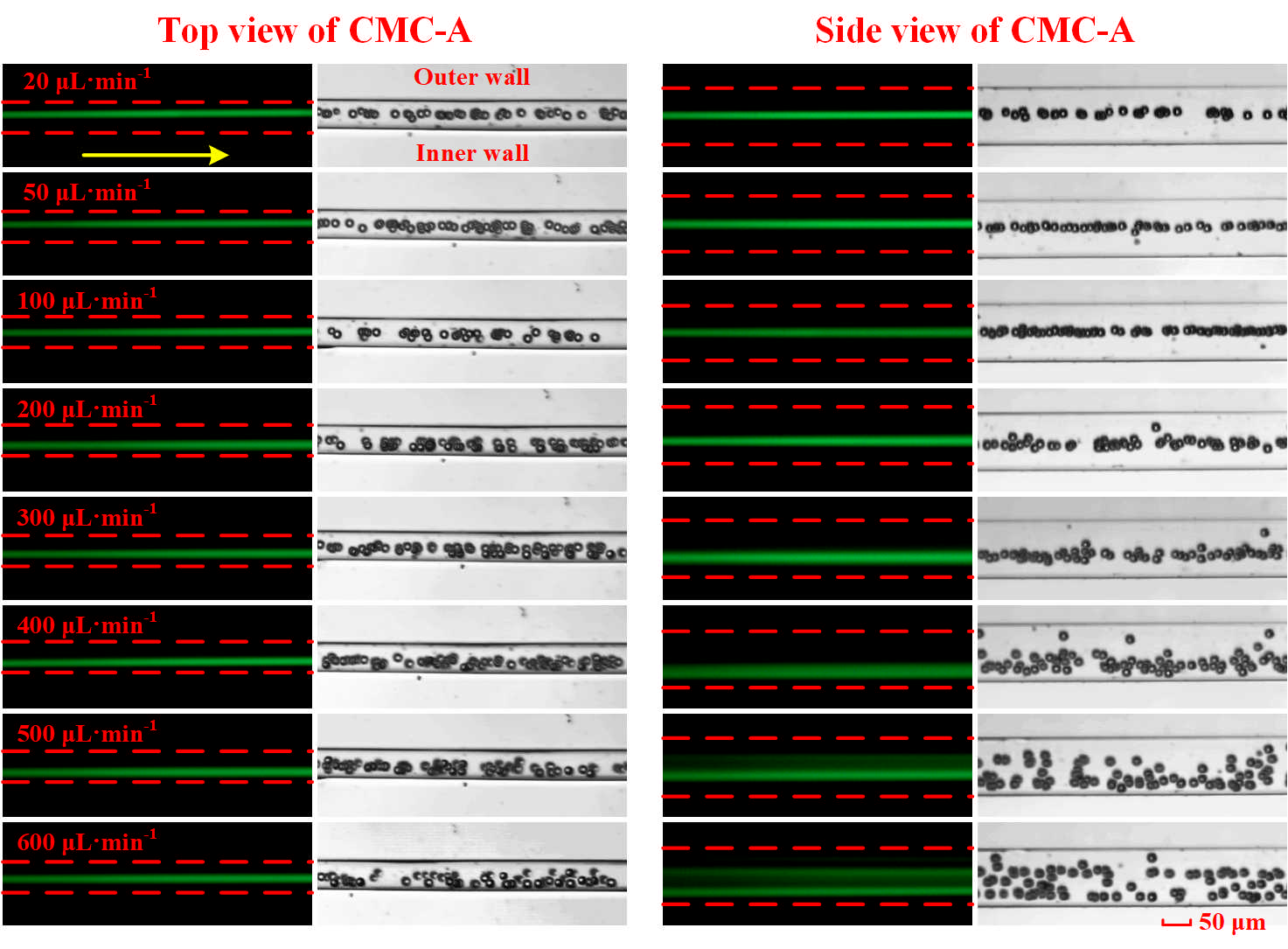


Fig. S6 Focusing behavior of 15 μm particles in CMC-A with 0.05 wt% HA-PBS solution. Dual-view (top and side) fluorescent images and high-speed camera images (stacked 100 frames) at flow rates ranging from 20 to 600 μL·min⁻¹. Yellow arrows indicate the flow direction and the red dashed lines represent the channel walls. Scale bar: 50 μm.


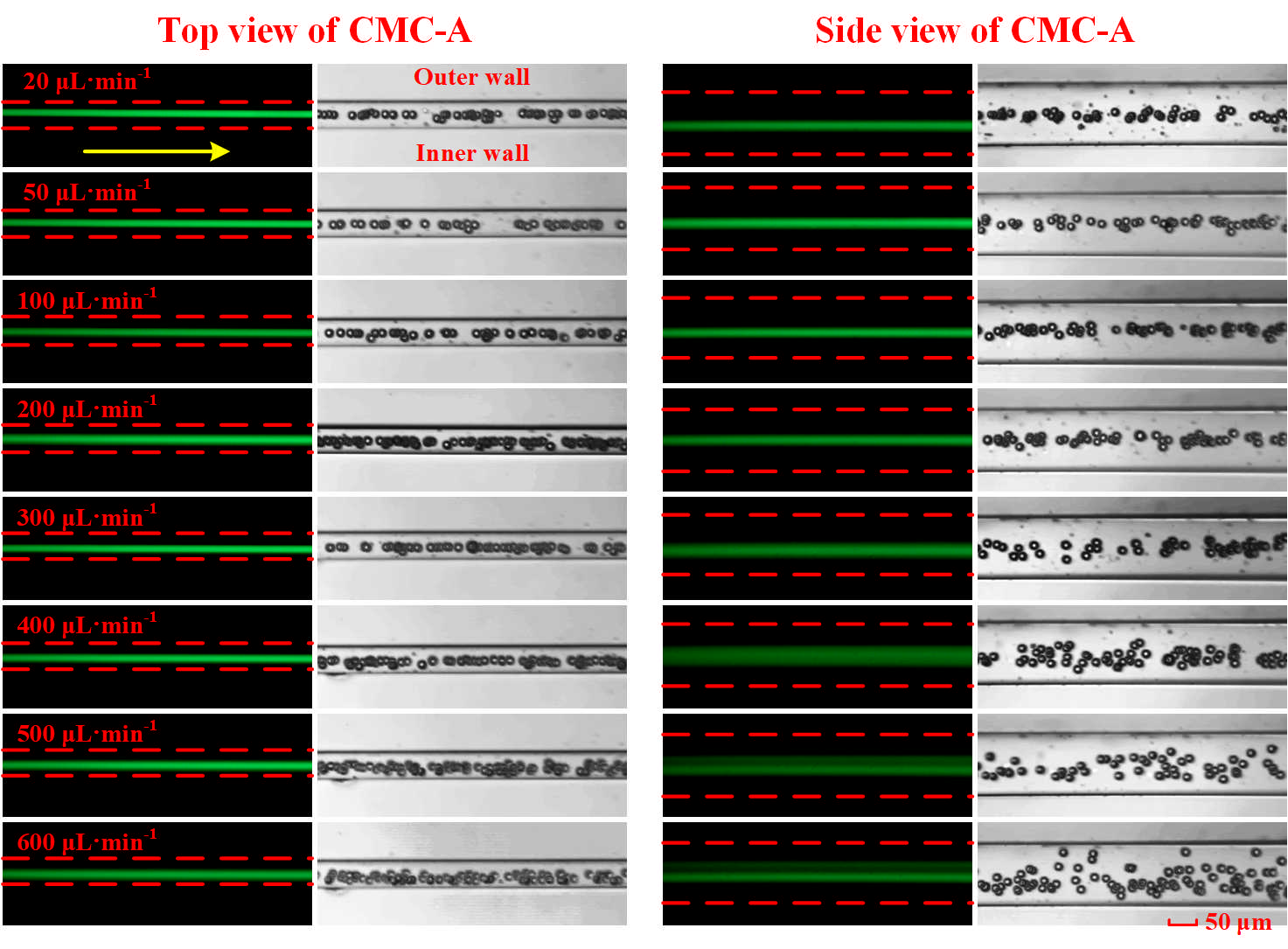


Fig. S7 Focusing behavior of 15 μm particles in CMC-A with 0.1 wt% HA-PBS solution. Dual-view (top and side) fluorescent images and high-speed camera images (stacked 100 frames) at flow rates ranging from 20 to 600 μL·min⁻¹. Yellow arrows indicate the flow direction and the red dashed lines represent the channel walls. Scale bar: 50 μm.


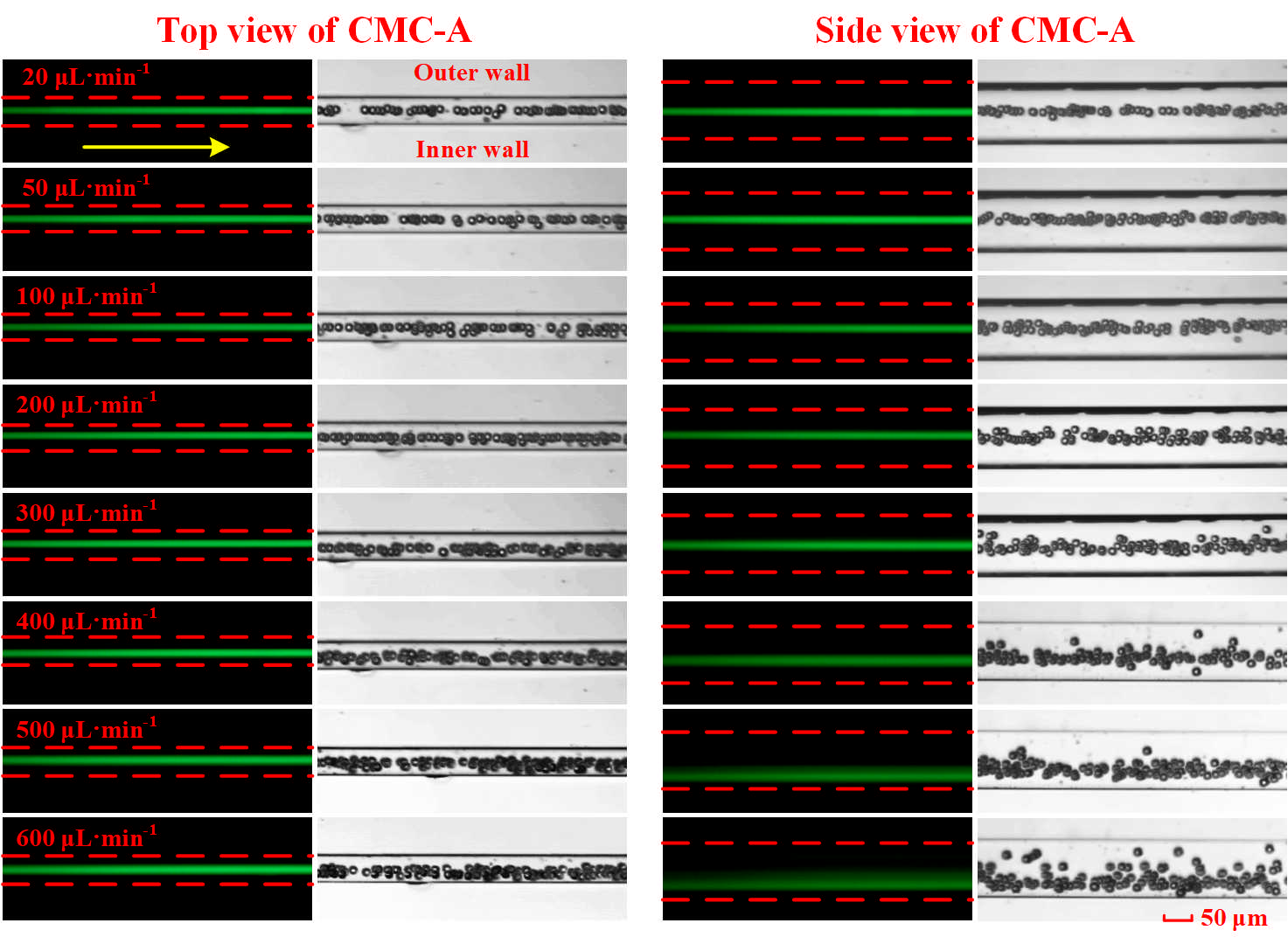


Fig. S8 Focusing behavior of 15 μm particles in CMC-A with 0.2 wt% HA-PBS solution. Dual-view (top and side) fluorescent images and high-speed camera images (stacked 100 frames) at flow rates ranging from 20 to 600 μL·min⁻¹. Yellow arrows indicate the flow direction and the red dashed lines represent the channel walls. Scale bar: 50 μm.


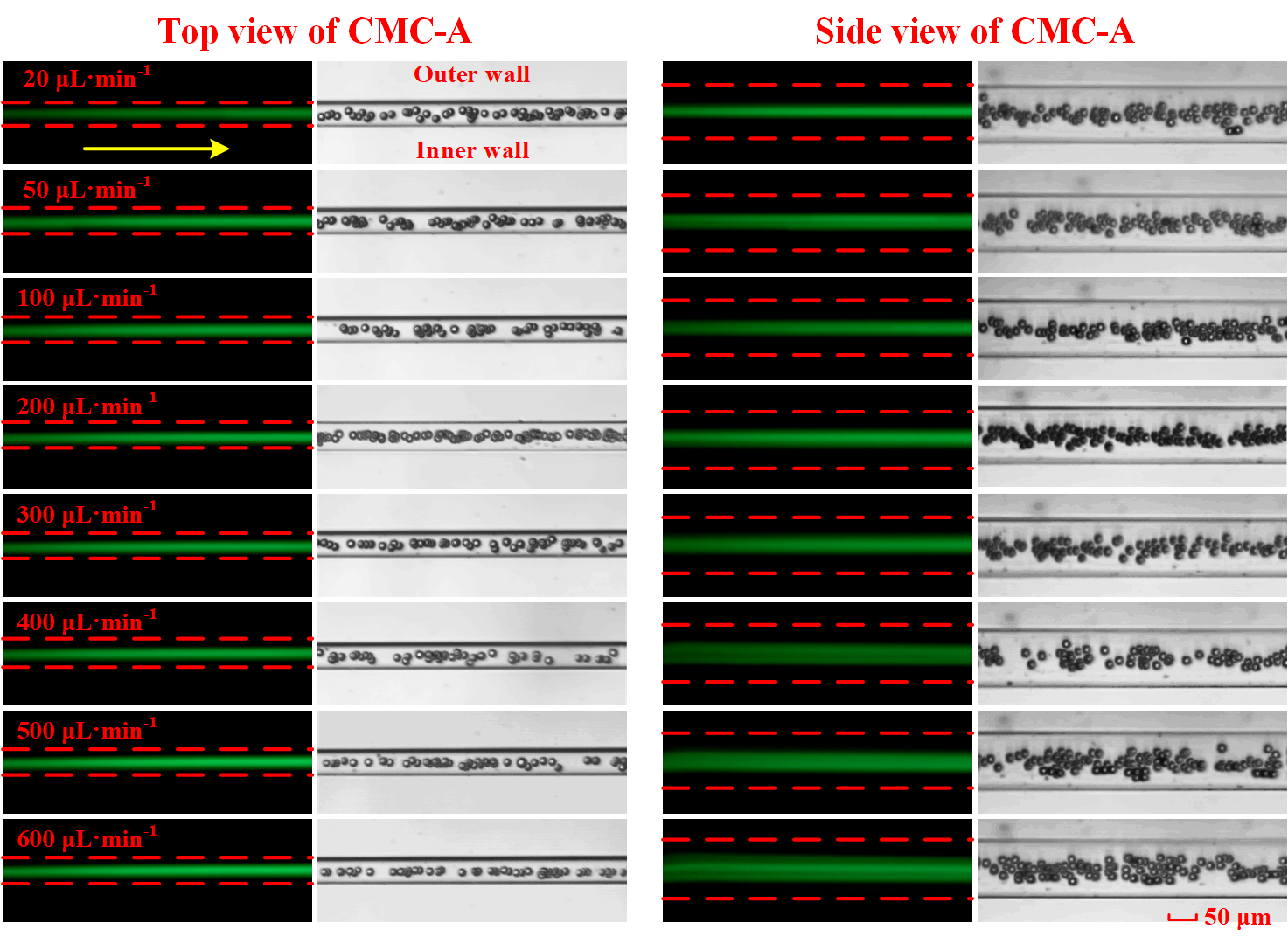


Fig. S9 Focusing behavior of 15 μm particles in CMC-A with 0.4 wt% HA-PBS solution. Dual-view (top and side) fluorescent images and high-speed camera images (stacked 100 frames) at flow rates ranging from 20 to 600 μL·min⁻¹. Yellow arrows indicate the flow direction and the red dashed lines represent the channel walls. Scale bar: 50 μm.


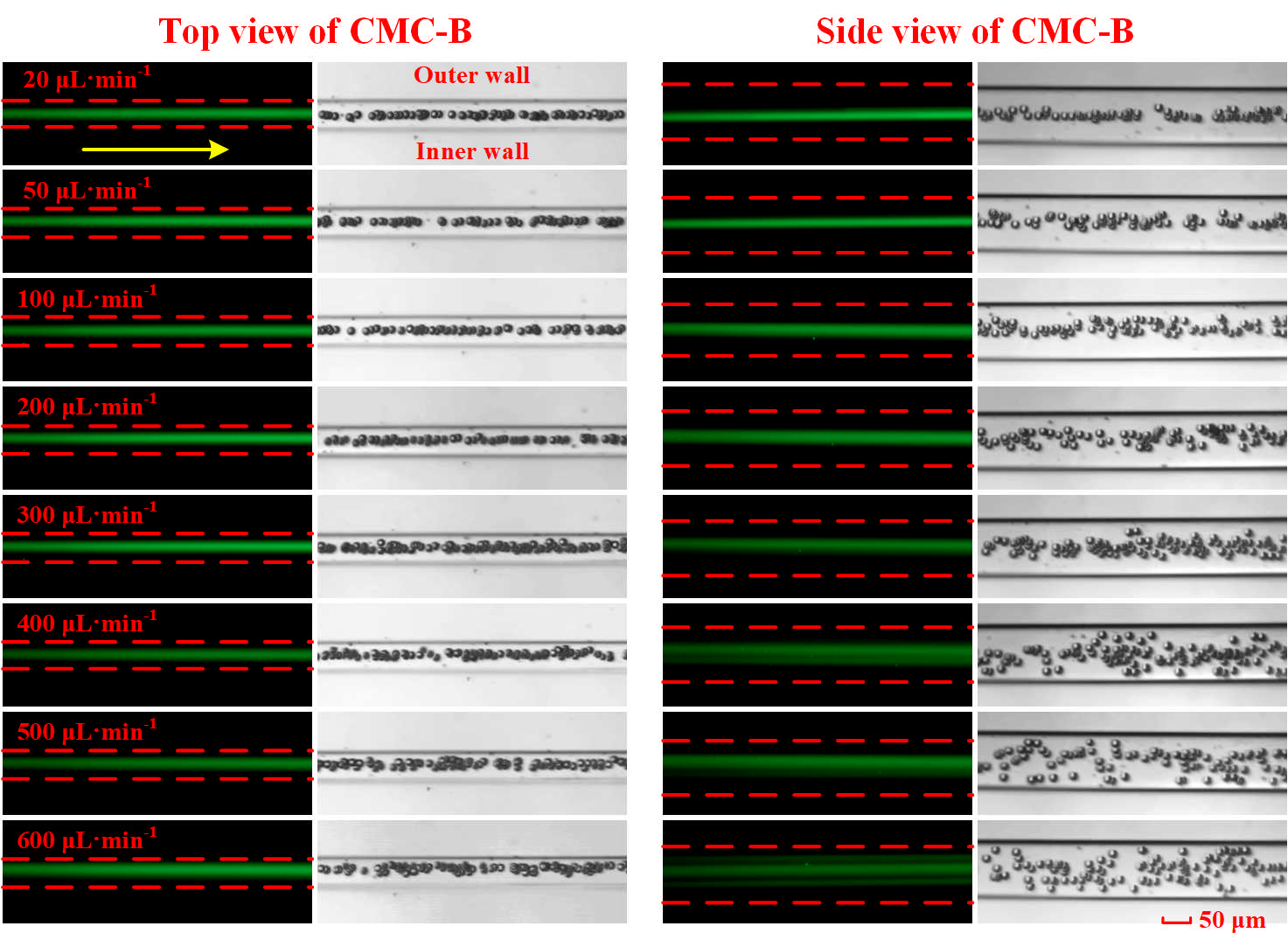


Fig. S10 Focusing behavior of 15 μm particles in CMC-B with 0.2 wt% HA-PBS solution. Dual-view (top and side) fluorescent images and high-speed camera images (stacked 100 frames) at flow rates ranging from 20 to 600 μL·min⁻¹. Yellow arrows indicate the flow direction and the red dashed lines represent the channel walls. Scale bar: 50 μm.


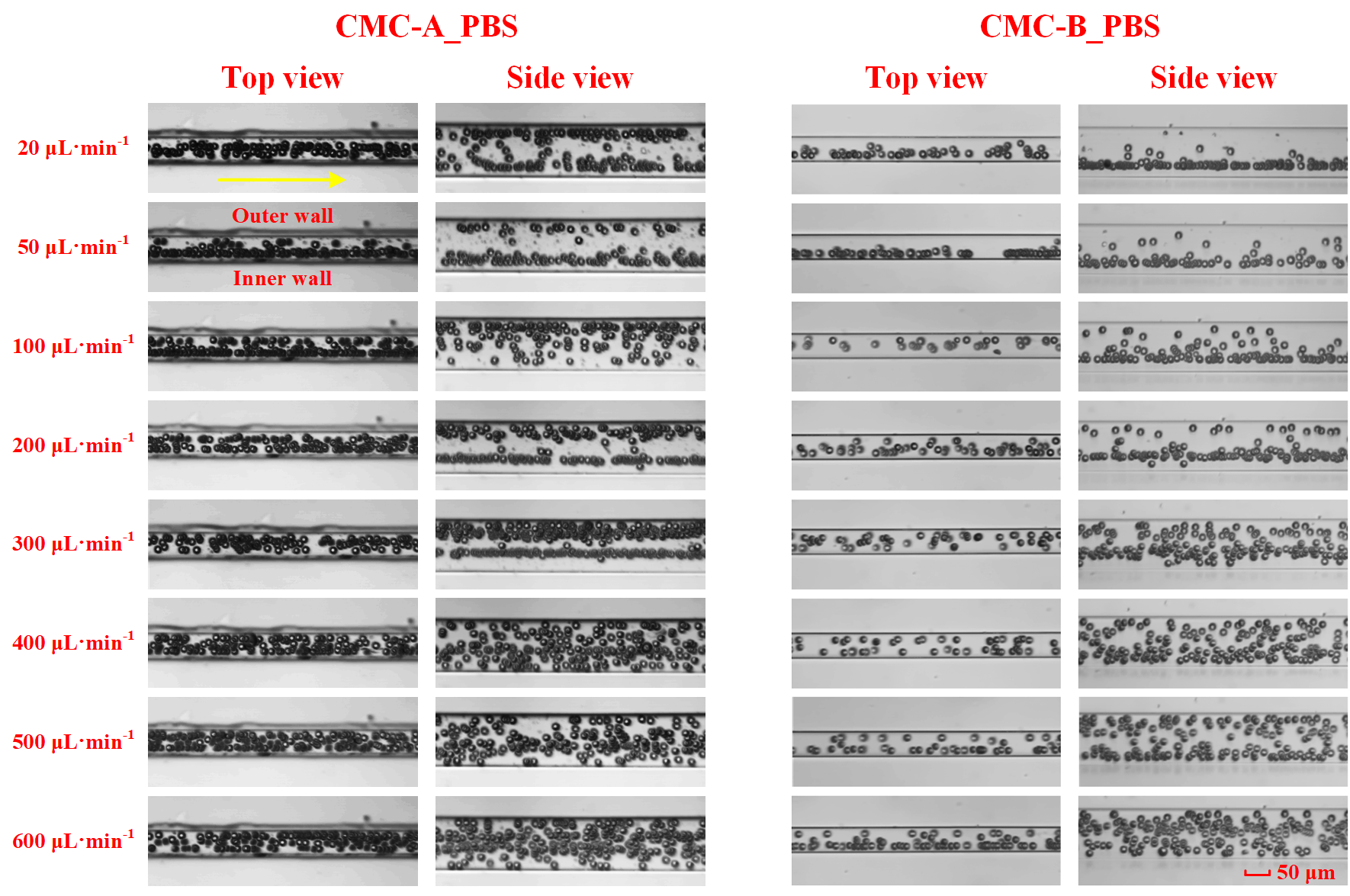


Fig. S11 Focusing behavior of 15 μm particles in CMC-A and CMC-B was examined using PBS solution. Dual-view (top and side) and high-speed camera images (stacked from 100 frames) were obtained at flow rates ranging from 20 to 600 μL·min⁻¹. Yellow arrows indicated the flow direction. Scale bar: 50 μm.


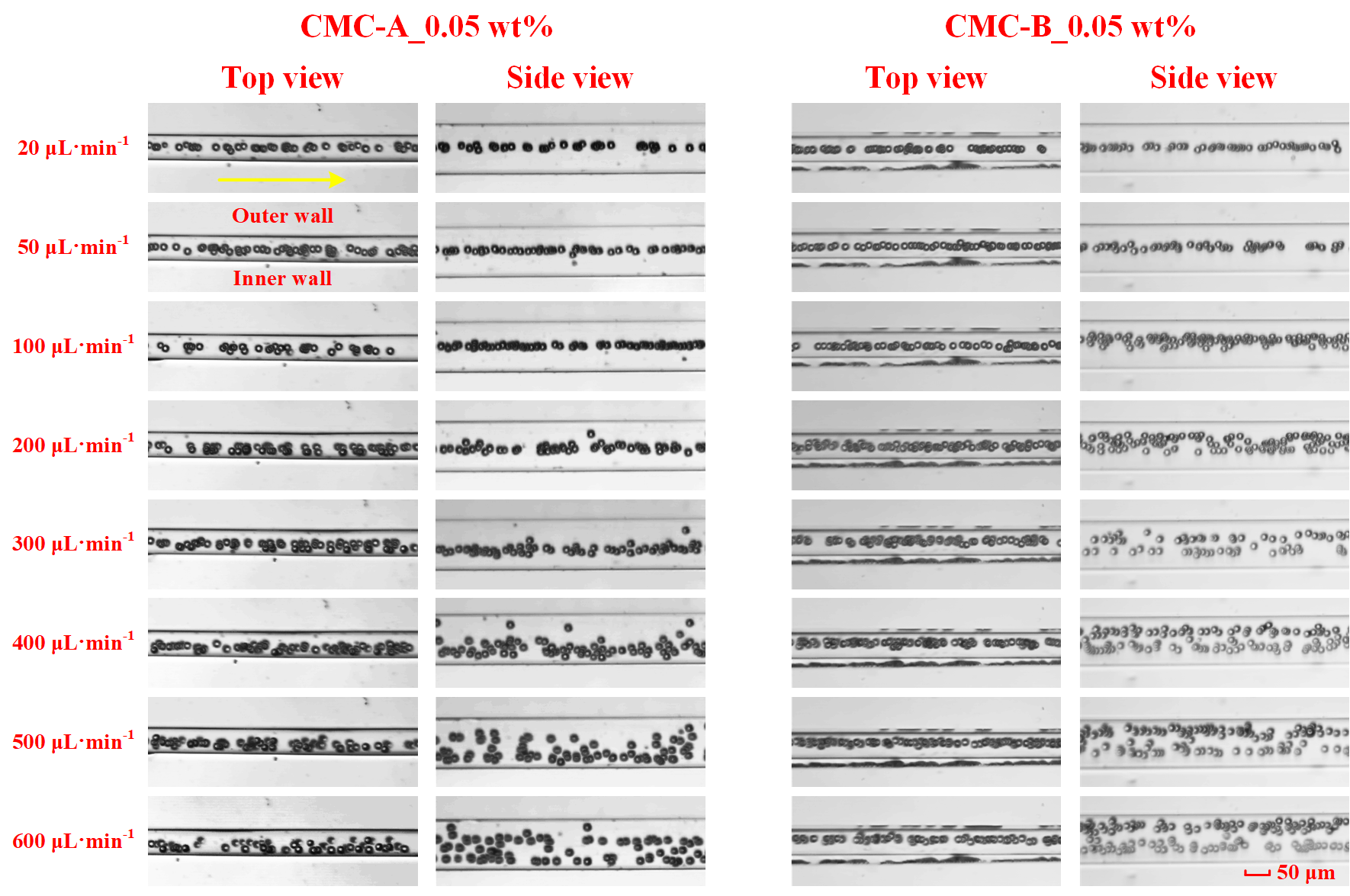


Fig. S12 Focusing behavior of 15 μm particles in CMC-A and CMC-B was examined using 0.05 wt% HA-PBS solution. Dual-view (top and side) and high-speed camera images (stacked from 100 frames) were obtained at flow rates ranging from 20 to 600 μL·min⁻¹. Yellow arrows indicated the flow direction. Scale bar: 50 μm.


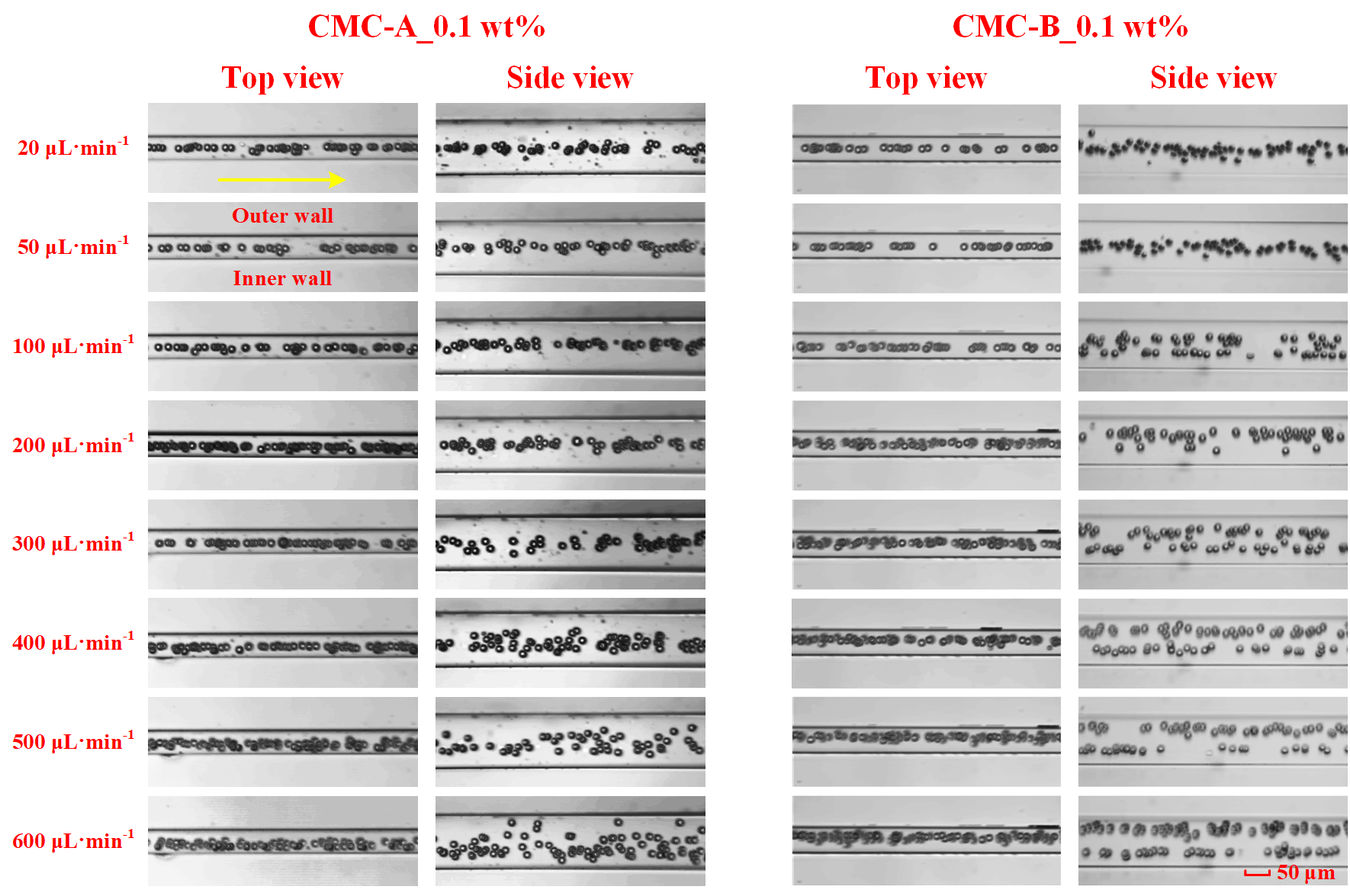


Fig. S13 Focusing behavior of 15 μm particles in CMC-A and CMC-B was examined using 0.1 wt% HA-PBS solution. Dual-view (top and side) and high-speed camera images (stacked from 100 frames) were obtained at flow rates ranging from 20 to 600 μL·min⁻¹. Yellow arrows indicated the flow direction. Scale bar: 50 μm.


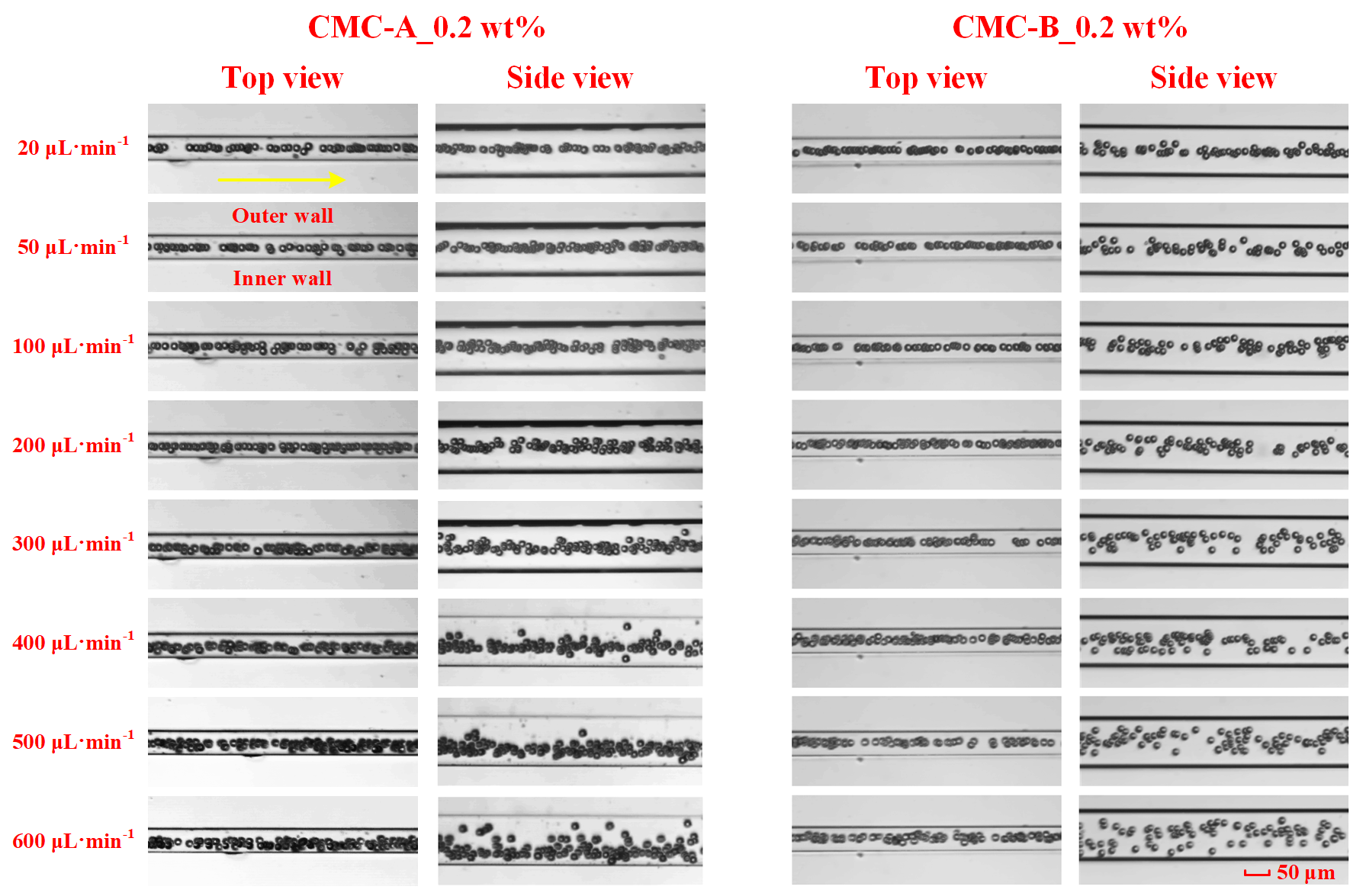


Fig. S14 Focusing behavior of 15 μm particles in CMC-A and CMC-B was examined using 0.2 wt% HA-PBS solution. Dual-view (top and side) and high-speed camera images (stacked from 100 frames) were obtained at flow rates ranging from 20 to 600 μL·min⁻¹. Yellow arrows indicated the flow direction. Scale bar: 50 μm.


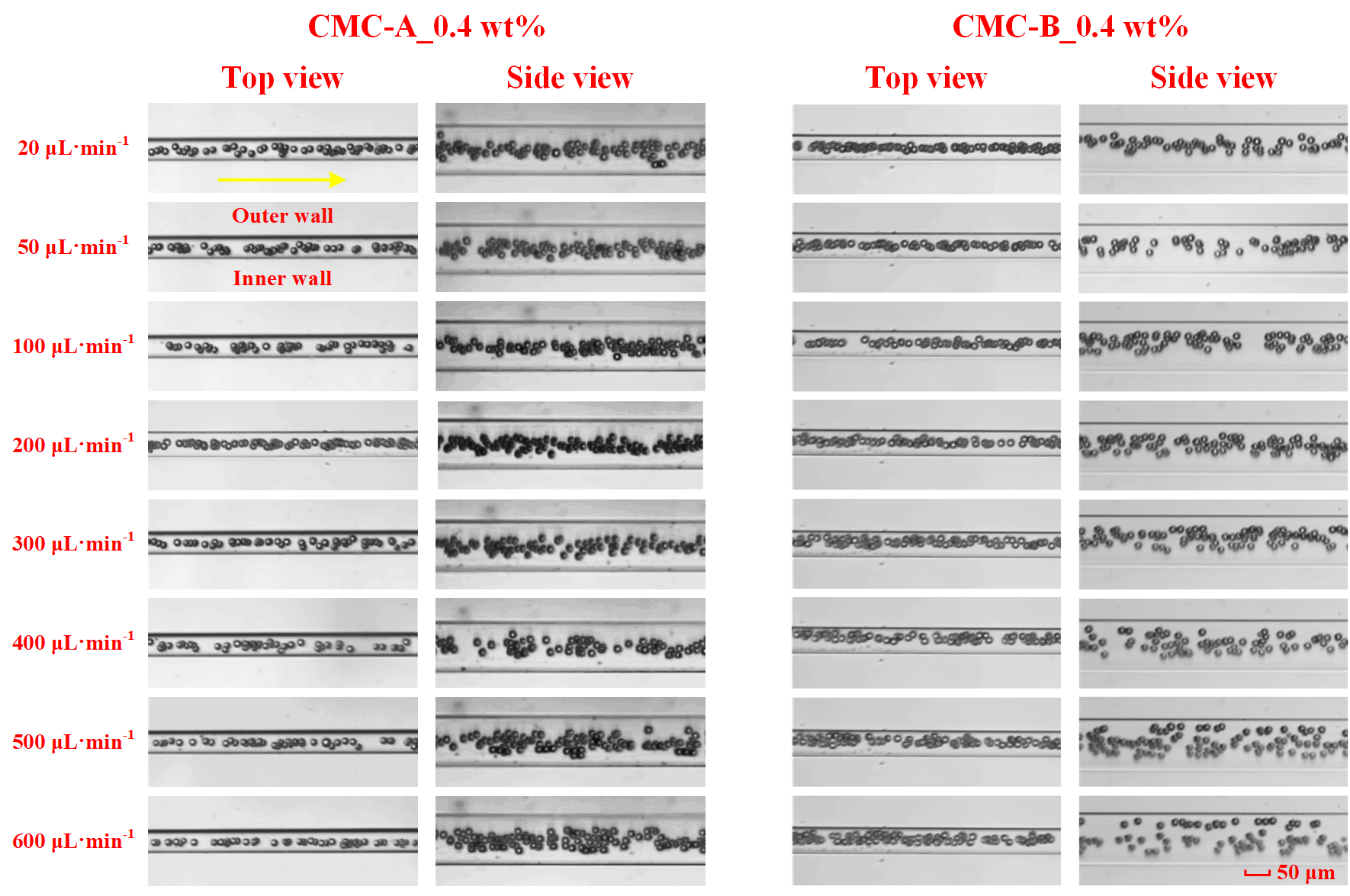


Fig. S15 Focusing behavior of 15 μm particles in CMC-A and CMC-B was examined using 0.4 wt% HA-PBS solution. Dual-view (top and side) and high-speed camera images (stacked from 100 frames) were obtained at flow rates ranging from 20 to 600 μL·min⁻¹. Yellow arrows indicated the flow direction. Scale bar: 50 μm.


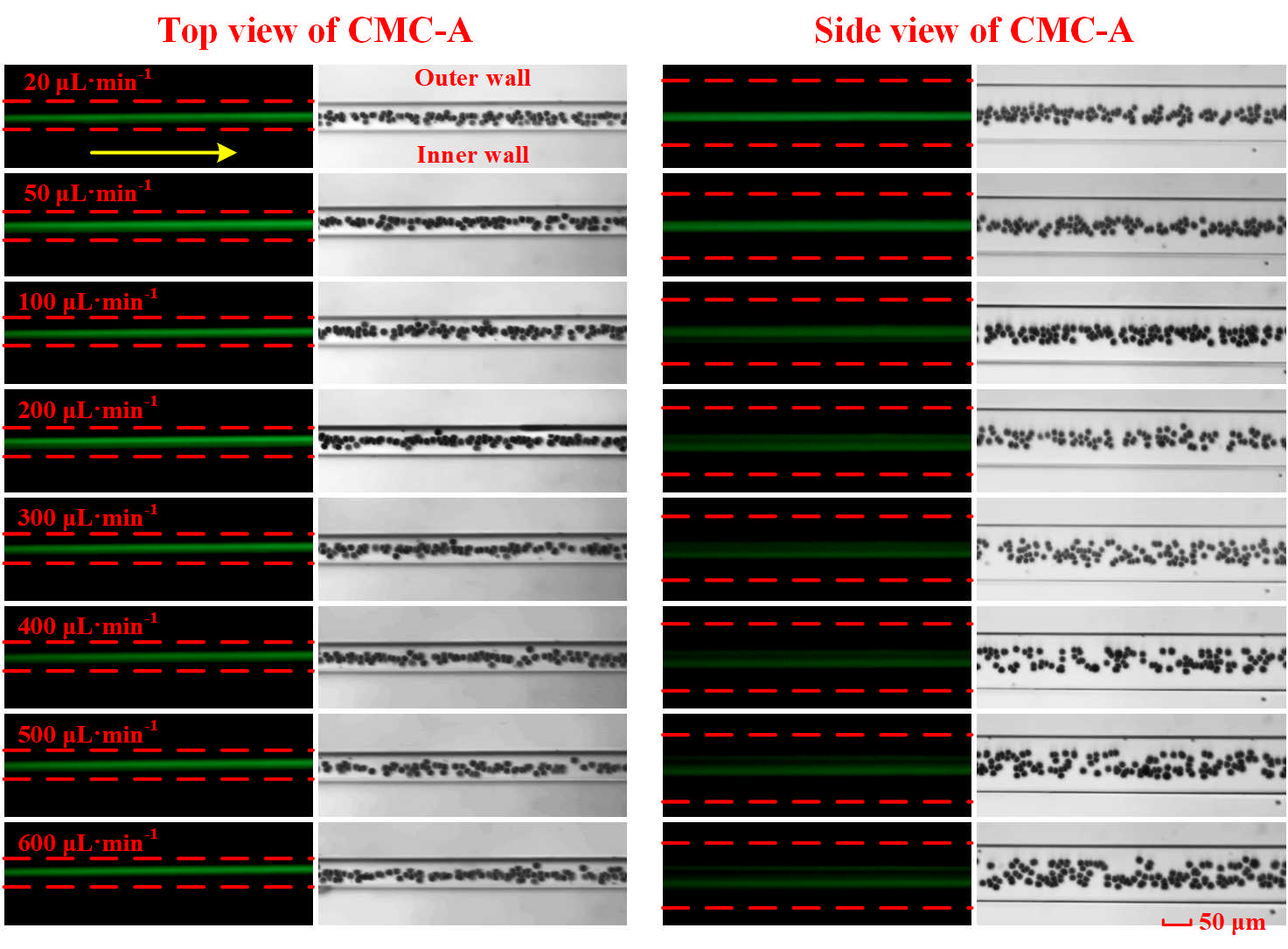


Fig. S16 Focusing behavior of 10 μm particles in CMC-A with 0.2 wt% HA-PBS solution. Dual-view (top and side) fluorescent images and high-speed camera images (stacked 100 frames) at flow rates ranging from 20 to 600 μL·min⁻¹. Yellow arrows indicate the flow direction and the red dashed lines represent the channel walls. Scale bar: 50 μm.


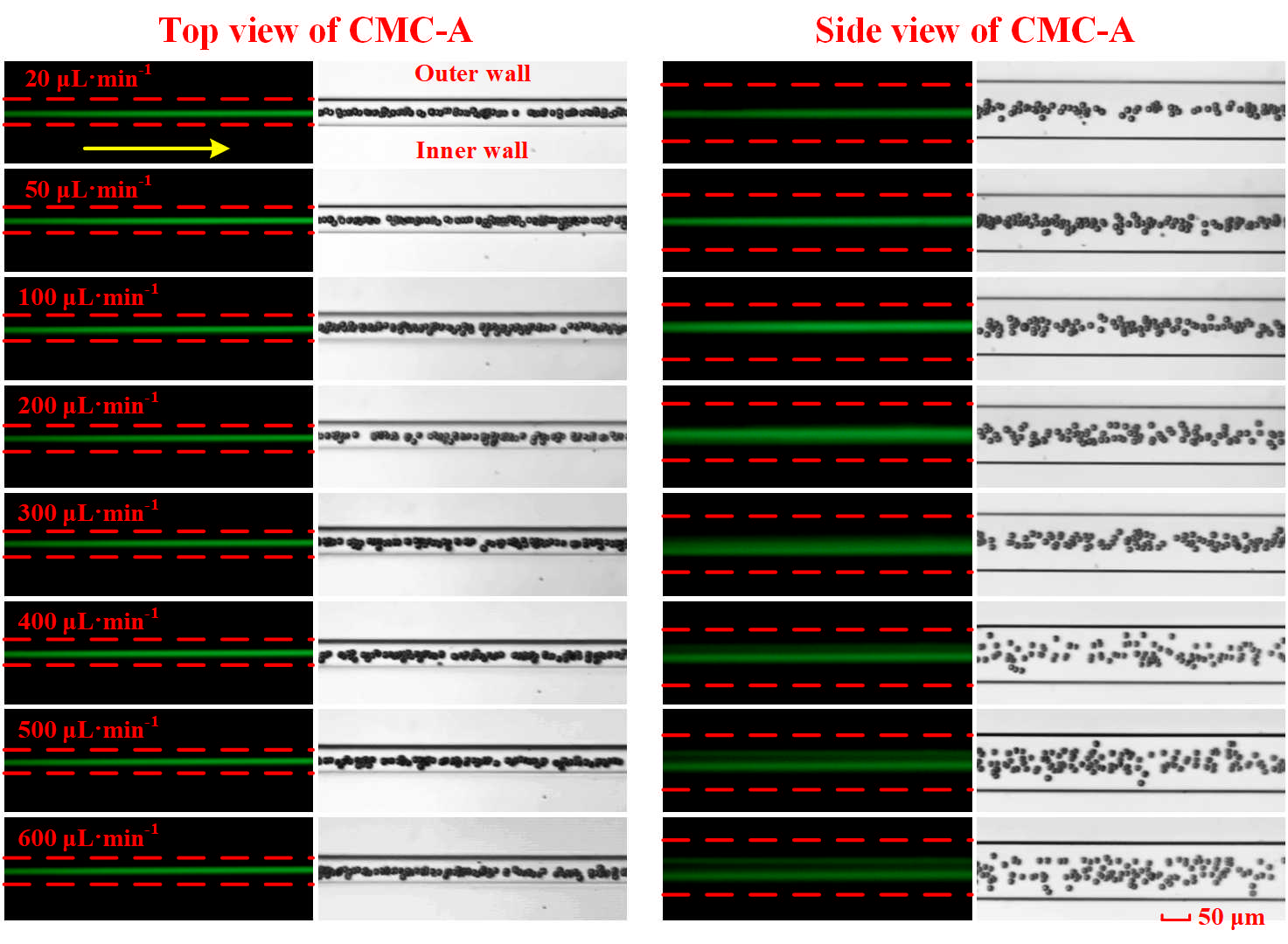


Fig. S17 Focusing behavior of 12 μm particles in CMC-A with 0.2 wt% HA-PBS solution. Dual-view (top and side) fluorescent images and high-speed camera images (stacked 100 frames) at flow rates ranging from 20 to 600 μL·min⁻¹. Yellow arrows indicate the flow direction and the red dashed lines represent the channel walls. Scale bar: 50 μm.


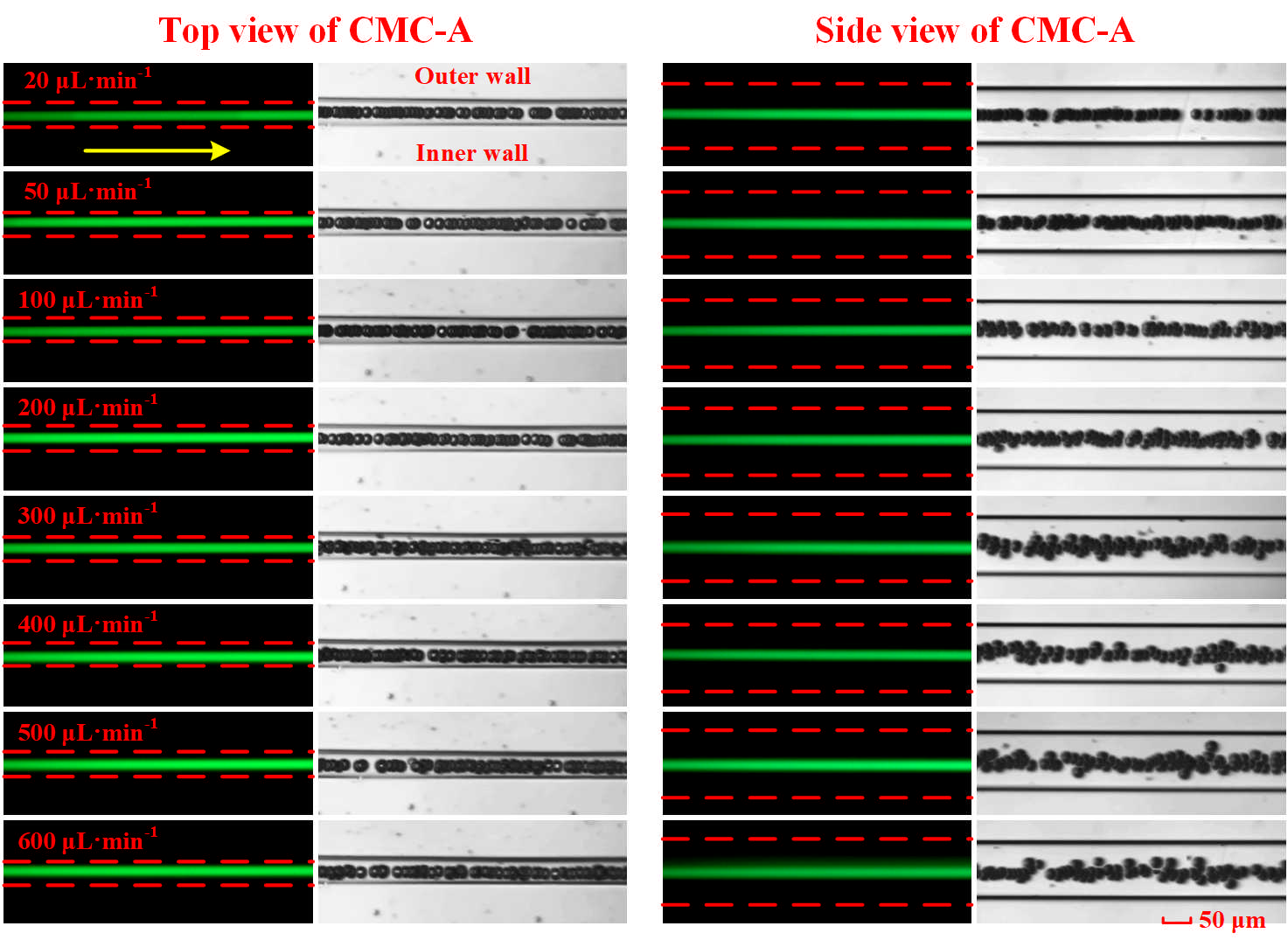


Fig. S18 Focusing behavior of 20 μm particles in CMC-A with 0.2 wt% HA-PBS solution. Dual-view (top and side) fluorescent images and high-speed camera images (stacked 100 frames) at flow rates ranging from 20 to 600 μL·min⁻¹. Yellow arrows indicate the flow direction and the red dashed lines represent the channel walls. Scale bar: 50 μm.


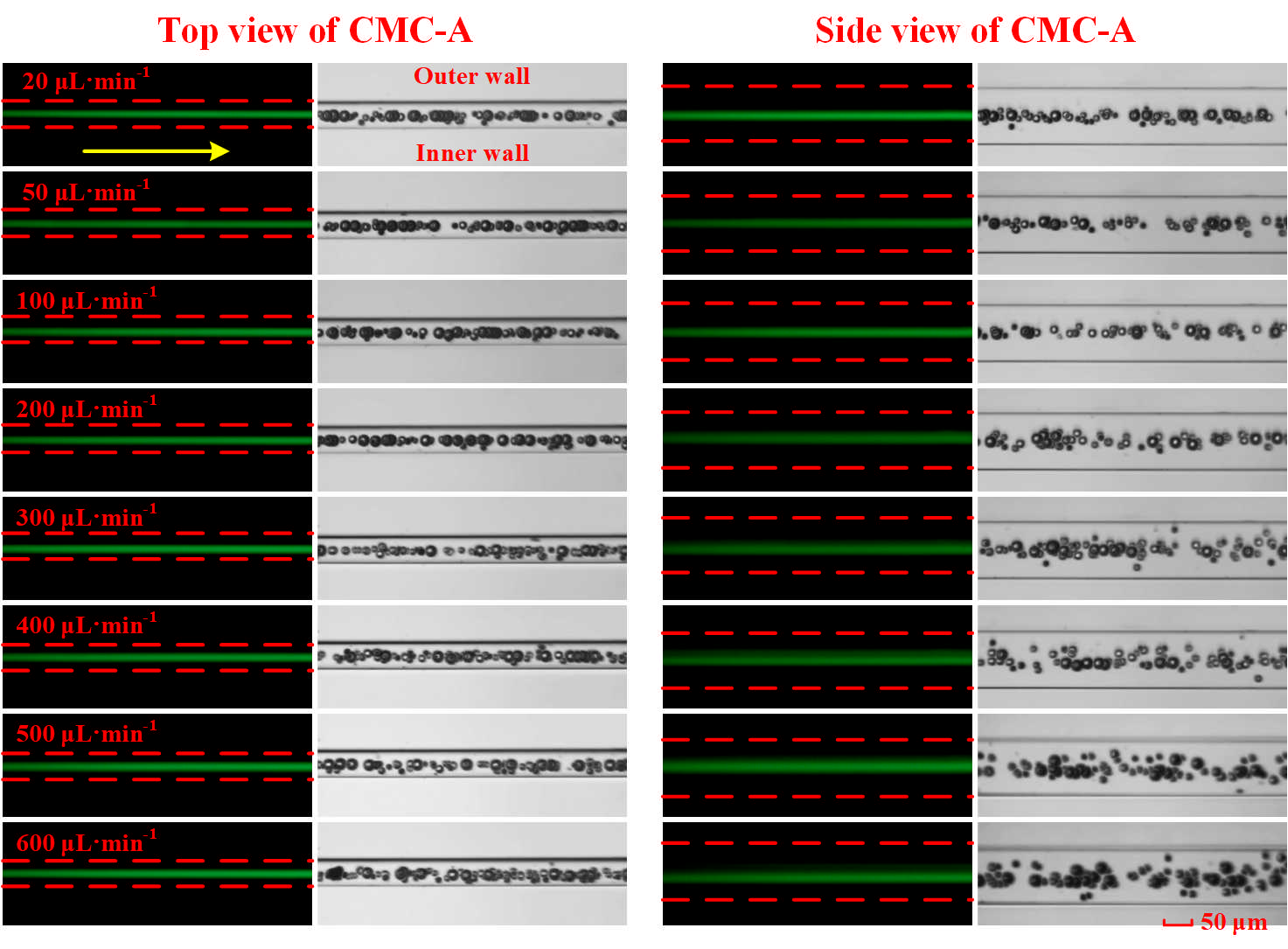


Fig. S19 Focusing behavior of particle mixtures (10, 12, 15, and 20 μm; 1:1:1:1 ratio) in CMC-A with 0.2 wt% HA-PBS solution. Dual-view (top and side) fluorescent images and high-speed camera images (stacked 100 frames) at flow rates ranging from 20 to 600 μL·min⁻¹. Yellow arrows indicate the flow direction and the red dashed lines represent the channel walls. Scale bar: 50 μm.


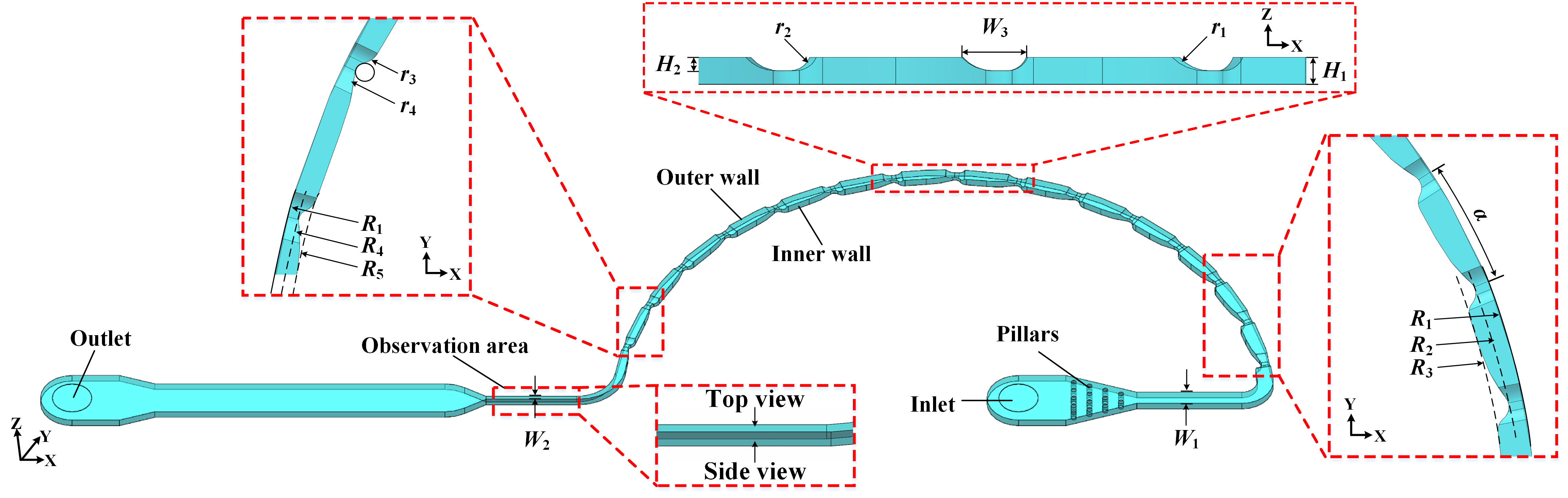


Fig. S20 The design of CMC-A. In-phase configuration: horizontal and vertical obstacles were spatially aligned.


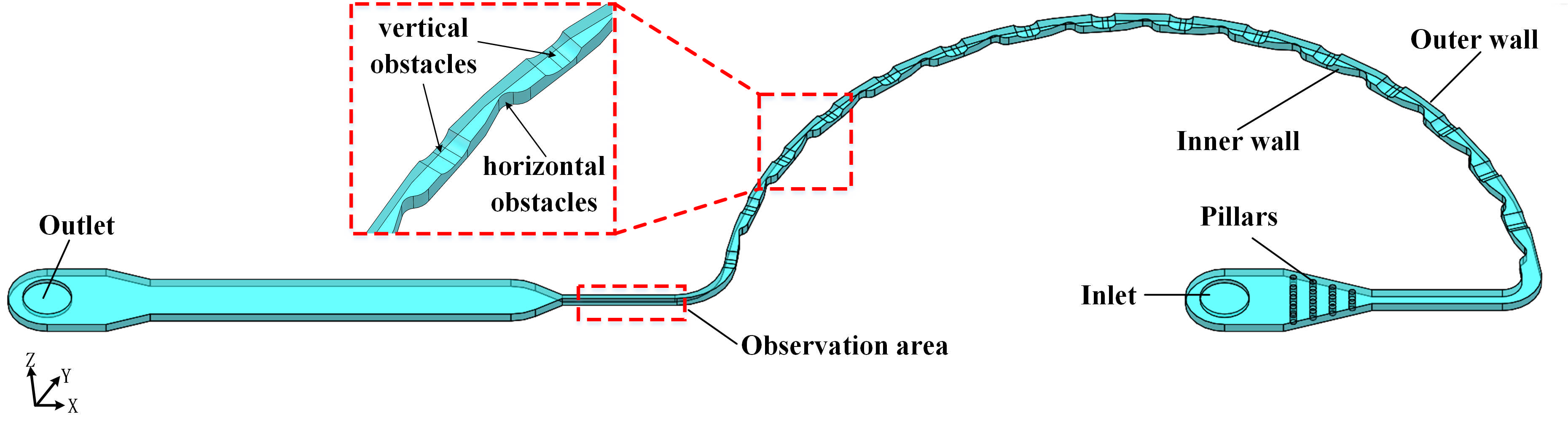


Fig. S21 The design of CMC-B. Out-of-phase configuration: horizontal obstacles were offset by 1/2 period relative to the vertical obstacles.


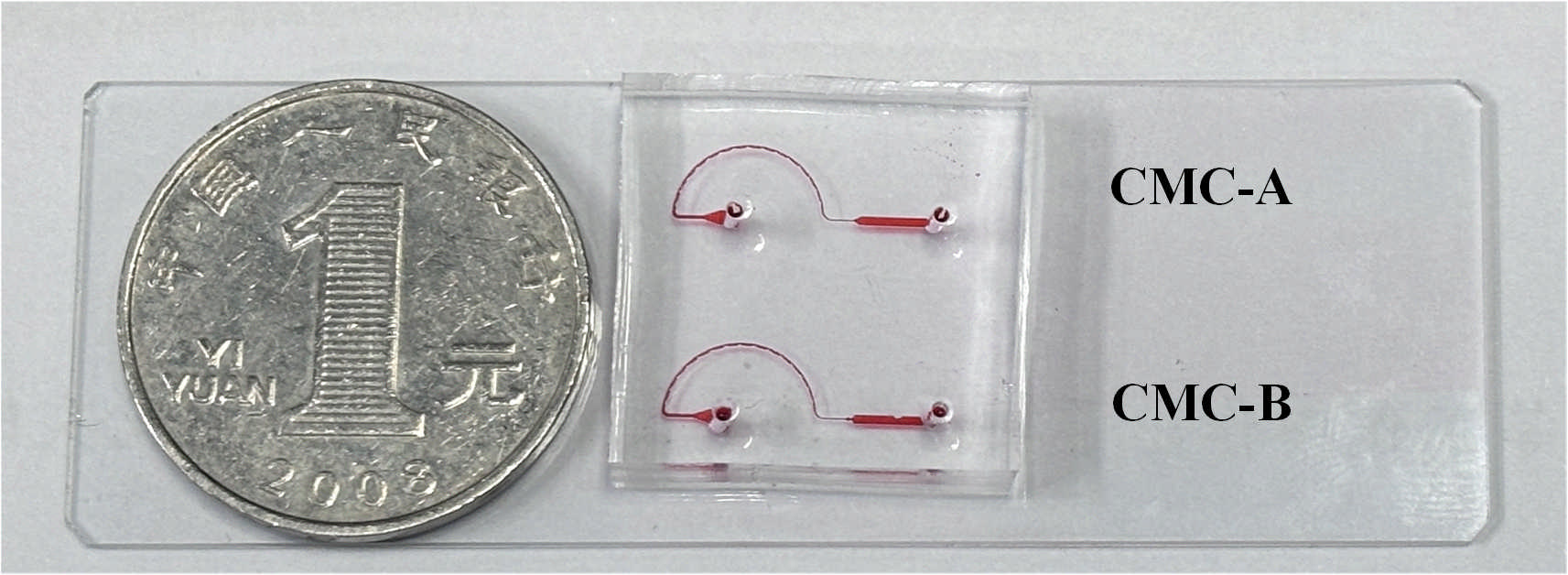


Fig. S22 The fabricated chips. CMC-A and CMC-B were injected with red ink to display the channel structure more clearly, and a 1-yuan Chinese coin was used as a reference.

1. F. Del Giudice *et al.*, Relaxation time of polyelectrolyte solutions: Whenμ-rheometry steps in charge. *J. Rheol.* **61**, 13-21 (2017).
